# Butyrate Protects Pancreatic Beta Cells from Cytokine-Induced Dysfunction

**DOI:** 10.3390/ijms221910427

**Published:** 2021-09-27

**Authors:** Michala Prause, Signe Schultz Pedersen, Violeta Tsonkova, Min Qiao, Nils Billestrup

**Affiliations:** 1 Department of Biomedical Sciences, University of Copenhagen, 2200 Copenhagen, Denmark; michalapr@sund.ku.dk (M.P.); signe.schultz.pedersen@sund.ku.dk (S.S.P.); vgts@novonordisk.dk (V.T.); min.qiao@uni-saarland.de (M.Q.); 2 Department of Stem Cell Discovery, Novo Nordisk A/S, 2760 Maaloev, Denmark

**Keywords:** insulin secretion, beta cell, butyrate, inflammation, cytokines

## Abstract

Pancreatic beta cell dysfunction caused by metabolic and inflammatory stress contributes to the development of type 2 diabetes (T2D). Butyrate, produced by the gut microbiota, has shown beneficial effects on glucose metabolism in animals and humans and may directly affect beta cell function, but the mechanisms are poorly described. The aim of this study was to investigate the effect of butyrate on cytokine-induced beta cell dysfunction in vitro. Mouse islets, rat INS-1E, and human EndoC-βH1 beta cells were exposed long-term to non-cytotoxic concentrations of cytokines and/or butyrate to resemble the slow onset of inflammation in T2D. Beta cell function was assessed by glucose-stimulated insulin secretion (GSIS), gene expression by qPCR and RNA-sequencing, and proliferation by incorporation of EdU into newly synthesized DNA. Butyrate protected beta cells from cytokine-induced impairment of GSIS and insulin content in the three beta cell models. Beta cell proliferation was reduced by both cytokines and butyrate. Expressions of the beta cell specific genes *Ins*, *MafA*, and *Ucn3* reduced by the cytokine IL-1β were not affected by butyrate. In contrast, butyrate upregulated the expression of secretion/transport-related genes and downregulated inflammatory genes induced by IL-1β in mouse islets. In summary, butyrate prevents pro-inflammatory cytokine-induced beta cell dysfunction.

## 1. Introduction

Impaired insulin secretion from pancreatic beta cells is characteristic for type 2 diabetes (T2D), and is the main cause of glucose intolerance. In the pathogenesis of T2D, it is hypothesized that during progression to diabetes, beta cells become dysfunctional and undergo changes into a less mature phenotype as a result of metabolic and inflammatory stress [1,2,3]. This decline in beta cell function is already present in individuals with impaired glucose tolerance and further progresses to severe dysfunction in T2D, without changes in beta cell volume [4]. The dysfunction is characterized by reduced glucose-stimulated insulin secretion (GSIS) capacity and insulin content, and is associated with decreased expression of several beta cell genes known to be important for proper beta cell function [5,6,7]. In addition, pancreata from organ donors with T2D compared to non-diabetic organ donors, showed a 3-fold increase in the number of islet cells that no longer produced any detectable amount of islet hormones. This indicates that an increased number of islet cells have undergone dedifferentiation into a non-functional islet cell phenotype [8].

The mechanisms underlying this dedifferentiation process are not known. However, since T2D is characterized by local low-grade inflammation in the islets of Langerhans, it has been hypothesized that inflammatory signals play a role in this process [6]. In particular, the pro-inflammatory cytokine IL-1β is known to induce beta cell dysfunction and apoptosis [9]. However, most published studies have used short-term exposure to high (cytotoxic) concentrations of IL-1β to induce dysfunction [10,11], which does not resemble the slow onset of beta cell dysfunction in the low-grade inflammation seen in T2D. We have recently shown that the long-term exposure of beta cells to low non-cytotoxic concentrations of pro-inflammatory cytokines such as IL-1β induces a state of dedifferentiation similar to what is observed in T2D [12]. This dysfunction is characterized by diminished GSIS, insulin content, decreased expression of several beta cell-specific genes and epigenetic changes. In particular, IL-1β induces a decrease in histone H3K27 acetylation and H3K4 methylation associated with the transcription start sites of beta cell specific genes [12]. These studies have led to the hypothesis that long-term exposure of beta cells to pro-inflammatory cytokines can induce dedifferentiation and dysfunction through changes in histone modifications, leading to decreased expression of key beta cell genes.

A major goal in diabetes research is to identify means to preserve or enhance beta cell function and thereby improve glucose homeostasis. In the past decade, the gut microbiota has emerged as an important regulator of glucose metabolism [13]. Among others, microbiota-derived metabolites including short chain fatty acids (SCFAs) have been shown to be important regulators of host inflammation and metabolism [14,15,16,17]. The precise mechanism by which the gut microbiota-derived metabolites affect metabolic health is not known, but SCFAs have been shown to directly affect beta cell function [18,19,20,21,22]. Bacteria produce SCFAs from the fermentation of dietary fiber, which may explain the association between high fiber intake and metabolic health [23]. Interestingly, it was recently reported in a large human study that increased production of the SCFA butyrate was associated with improved insulin response after an oral glucose tolerance test [24]. This suggests that butyrate directly affects beta cells and somehow prevents dysfunction. In individuals with T2D, a significant decrease in butyrate-producing bacteria is observed [25]. A positive effect of butyrate has also been reported in diabetic mice [16,17,26,27,28,29], where administration of butyrate lowered blood glucose, increased plasma insulin, and reduced levels of circulating inflammatory cytokines. However, the mechanisms of action are poorly understood at present. While there is ample evidence for the importance of butyrate in metabolic health and glucose metabolism, its exact mechanism and targets are unknown. We here report the in vitro effect and underlying pathways of butyrate on cytokine-induced beta cell dysfunction, mimicking the beta cell fate during the pathogenesis of T2D.

## 2. Results

### 2.1. Butyrate Prevents IL-1β-Induced Impairment of Glucose-Stimulated Insulin Secretion and Insulin Content in Mouse Islets

**Figure 1:**
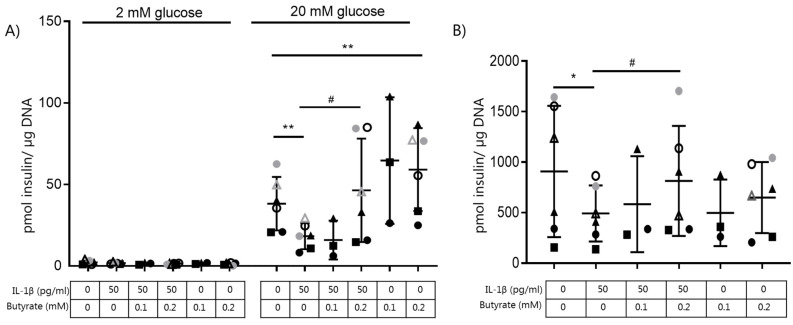
Butyrate prevents IL-1β-induced impairment of glucose-stimulated insulin secretion and content in mouse islets. Mouse islets were cultured for 10 days in the absence or presence of 50 pg/mL IL-1β and/or butyrate (0.1, 0.2 mM). (**A**) Insulin secretion was measured by static batch incubations for 30 min in response to 2 mmol/L glucose followed by 20 mmol/L and normalized to DNA content. (**B**) Total insulin content was measured post-glucose stimulation. Data are shown as means ± SD for *n* = 3–6 and analyzed by a 2-sided paired *t*-test. * *p* < 0.05, ** *p* < 0.01 vs. control, # *p* < 0.05 vs. IL-1β.

The effect of butyrate on IL-β-induced dysfunction in beta cells was evaluated by analyzing GSIS and insulin content in mouse islets exposed to combinations of IL-1β (50 pg/mL) and butyrate (0.1–0.2 mM) (Figure 1A,B) for 10 days. As shown previously [12], IL-1β significantly inhibited GSIS and decreased insulin content. Exposure of the islets to 0.2 mM butyrate, but not 0.1 mM butyrate, prevented the detrimental effects of IL-1β on beta cell insulin secretion (Figure 1A) and content (Figure 1B). Butyrate (0.2 mM) alone significantly enhanced GSIS compared to non-exposed control islets. Higher concentrations of butyrate (0.4–0.6 mM) did not further increase GSIS.

Apoptosis, measured as released DNA histone complexes from the nucleus to the cytosol, was not induced by 50 pg/mL IL-1β or 0.2 mM butyrate or the combination compared to non-exposed islets. As a positive control, we included islets exposed to a cytotoxic concentration of 300 pg/mL IL-1β for 48 h (Supplementary Figure S1).

### 2.2. Butyrate Prevents Cytokine-Induced Impairment of Glucose-Stimulated Insulin Secretion in Human EndoC-βH1 and Rat INS-1E Beta Cells

**Figure 2:**
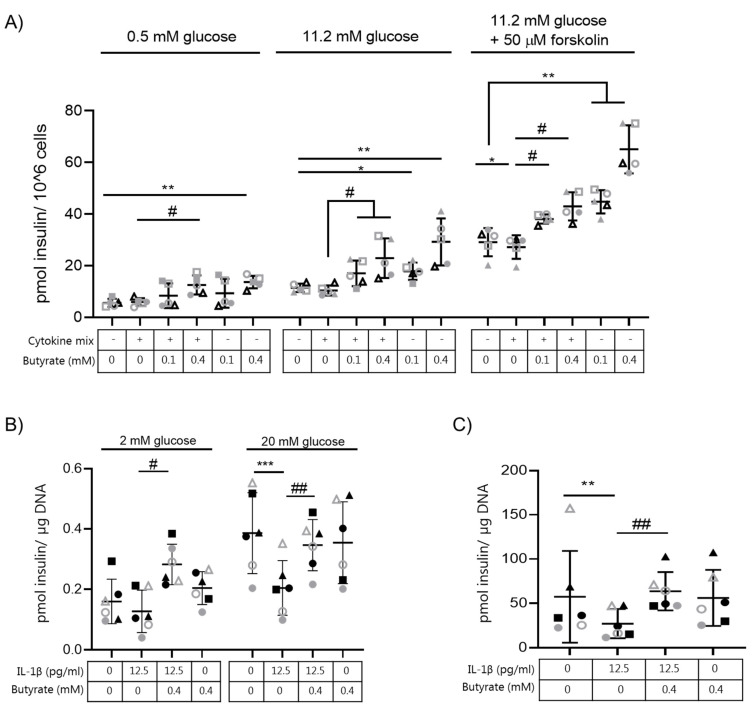
Butyrate prevents cytokine induced impairment of glucose-stimulated insulin secretion in human EndoC-βH1 cells and rat INS-1E cells. (**A**) Human EndoC-βH1 cells were cultured for 7 days in the absence or presence of the cytokine mixture (500 pg/mL IL-1β + 2 ng/mL INF-γ + 2 ng/mL TNF-α) and/or butyrate (0.1 mM or 0.4 mM). Insulin secretion was measured by static batch incubations for 45 min in response to 0.5 mmol/L glucose, 11.2 mmol/L glucose followed by 11.2 mmol/L glucose plus 50 µM forskolin and normalized to cell number. Each condition was tested in quadruplicate. Data are shown as means ± SD for *n* = 5 and analyzed by a 2-sided paired *t*-test. * *p* < 0.05, ** *p* < 0.01 vs. control, # *p* < 0.05 vs. cytokine mixture. (**B**) INS-1E cells were cultured for 3 days in the absence or presence of 12.5 pg/mL IL-1β and/or 0.4 mM butyrate. Insulin secretion was measured by static batch incubations for 30 min in response to 2 mmol/L glucose followed by 20 mmol/L and normalized to DNA content. (**C**) Total insulin content was measured post-glucose stimulation. Data are shown as means ± SD for *n* = 6 and analyzed by a 2-sided paired *t*-test. ** *p* < 0.01, *** *p* < 0.001 vs. control, ## *p* < 0.01 vs. IL-1β.

Next, we investigated the effect of butyrate on insulin secretion in other beta cell models. We therefore tested different doses of IL-1β (200–500 pg/mL) combined with INF-γ (2 ng/mL) and TNF-α (2 ng/mL) in the human EndoC-βH1 cell line to determine the combination of cytokines needed to impair insulin secretion (Supplementary Figure S2A) without inducing apoptosis (Supplementary Figure S2B). Since EndoC-βH1 cells show a modest stimulation of insulin release by glucose alone, we included stimulation by forskolin in order to examine a more robust stimulation of secretion. Exposure of human EndoC-βH1 cells to IL-1β (500 pg/mL), INF-γ (2 ng/mL), and TNF-α (2 ng/mL) (cytokine mix) for 7 days significantly diminished GSIS when cells were challenged with 11.2 mM glucose plus 50 μM forskolin (Figure 2A, Supplementary Figure S2A) compared to non-exposed control cells. The chosen cytokine mix did not induce apoptosis in EndoC-βH1 cells but induced a small, yet significant, increase in caspase 3/7 positive cells (Supplementary Figure S2C).

We next exposed EndoC-βH1 cells to butyrate (0.1 or 0.4 mM) in combination with the cytokine mix. The cytokine-induced reduction in insulin secretion in response to 11.2 mM glucose and 50 μM forskolin was abolished when the cells were co-exposed to butyrate (0.1 and 0.4 mM) (Figure 2A). Exposure to butyrate alone also boosted insulin secretion in EndoC-βH1 cells compared to non-exposed cells when cells were challenged with either 2 mM or 11.2 mM glucose alone, or combined with 50 μM forskolin (Figure 2A).

We also tested the effect of butyrate (0.4 mM) on insulin-secreting INS-1E cells exposed to IL-1β for 3 days. The minimal dose of IL-1β (12.5 pg/mL) required to induce dysfunctional INS-1E cells (reduced GSIS and insulin content) without affecting apoptosis was determined by a dose curve experiment (Supplementary Figure S3A–C). Again, butyrate abrogated the negative effect of IL-1β on GSIS (Figure 2B) and reestablished insulin content to control level (Figure 2C). Exposure to butyrate alone affected neither GSIS nor insulin content compared to non-exposed INS-1E cells.

Together, these results show that butyrate significantly prevents impaired GSIS and insulin content induced by non-cytotoxic long-term exposure of IL-1β or cytokines in three different beta cell models. Furthermore, insulin secretion was significantly elevated by butyrate alone when compared to the non-exposed mouse islets and the human EndoC-βH1 beta cell line.

### 2.3. Butyrate Blocks Beta Cell Proliferation in Mouse Islets and EndoC-βH1 Cells

**Figure 3:**
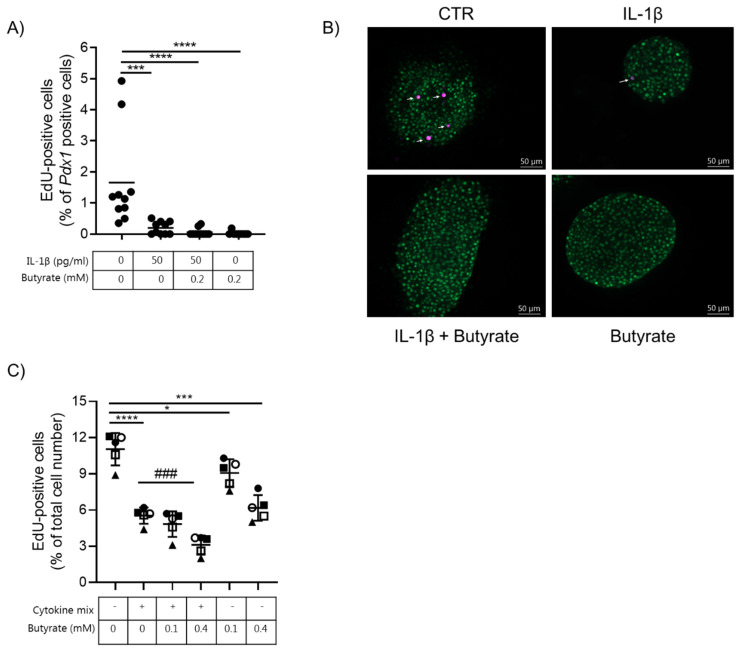
Butyrate and cytokines reduce mouse islet and human EndoC-βH1 beta cell proliferation. (**A**) Beta cell proliferation was examined in mouse islets exposed to 50 pg/mL IL-1β and/or 0.2 mM butyrate, or left non-exposed for 10 days. Proliferation was determined by staining for Pdx1 and EdU. Results are shown as percentage proliferating beta cells of total Pdx1 positive cells. Data are shown for *n* = 10 islets from three independent experiments and analyzed by an unpaired *t*-test. *** *p* < 0.001, **** *p* < 0.0001 vs. control. (**B**) Immunocytochemical staining of mouse islets. Cells were stained for Pdx1 (green) and EdU (magenta). Data shown are representative. (**C**) Beta cell proliferation was measured in EndoC-βH1 cells exposed to cytokine mix (500 pg/mL IL-1β + 2 ng/mL INF-γ + 2 ng/mL TNF-α), 0.1 or 0.4 mM butyrate, or left non-exposed for 6.5 days. Proliferation was measured as EdU-positive cells and shown as the percentage proliferating beta cells of the total cell number per well. Data are shown as means ± SD for *n* = 5 and analyzed by an unpaired *t*-test * *p* < 0.05, *** *p* < 0.001, **** *p* < 0.0001 vs. control, ### *p* < 0.001 vs. IL-1β. Each condition was tested in biological triplicate.

Beta cell proliferation was determined by EdU incorporation into *Pdx1*-positive cells. Intact mouse islets were exposed to 50 pg/mL IL-1β, 0.2 mM butyrate, or left un-exposed for 10 days. Under basal conditions, the beta cell proliferation rate was determined to be 1.7% (Figure 3A,B). The proliferation rate dropped significantly to 0.2%, 0.06%, and 0.02% in islets exposed to IL-1β, IL-1β and butyrate or butyrate alone, respectively. Proliferation data from the EndoC-βH1 cell line showed a similar trend: basal proliferation was significantly reduced by low non-cytotoxic doses of cytokines, 0.1 or 0.4 mM butyrate alone, or the combination with cytokines (Figure 3C).

### 2.4. Butyrate Does Not Affect Beta Cell Mature Gene Expression

**Figure 4:**
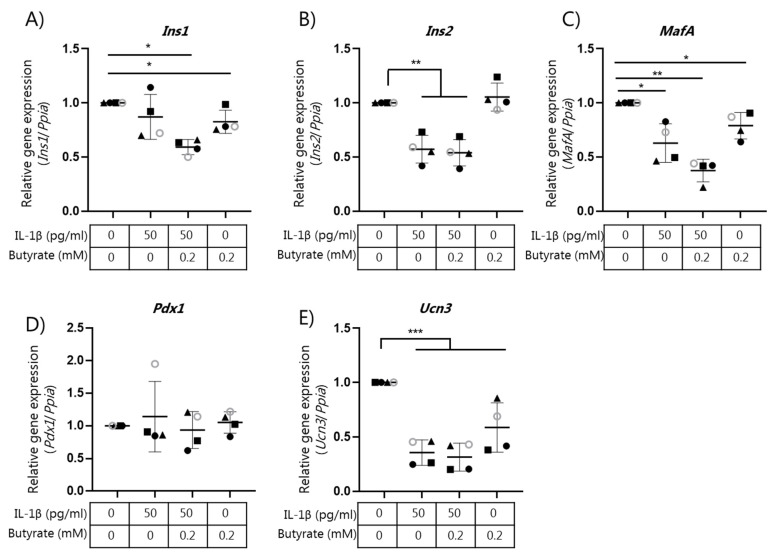
Expression of beta cell genes in mouse islets exposed to IL-1β and butyrate. Mouse islets were cultured for 10 days in the absence or presence of 50 pg/mL IL-1β and/or 0.2 mM butyrate. Relative mRNA expression of (**A**) *Ins1*, (**B**) *Ins2*, (**C**) *MafA*, (**D**) *Pdx1*, and (**E**) *Ucn3* were analyzed using qPCR. Expression levels were normalized to the expression of *Ppia* and control islet expression data were normalized to 1. Data are shown as means ± SD for *n* = 4 and analyzed by a 2-sided paired *t*-test. * *p* < 0.05, ** *p* < 0.01, *** *p* < 0.001 vs. control.

We have recently shown that IL-1β-mediated beta cell dysfunction is associated with global gene expression changes, and in particular by changes in the expression of genes associated with mature beta cell function [12]. We therefore analyzed if the effect of butyrate on insulin secretion was associated with changes in the expression of beta cell maturity genes. We examined gene expression by qPCR of the selected genes *Ins1*, *Ins2*, *MafA*, *Pdx1*, and *Ucn3* in mouse islets (Figure 4A–E). IL-1β (50 pg/mL) significantly reduced the expression of *Ins2* (Figure 4B), *MafA* (Figure 4C), and *Ucn3* (Figure 4E) in the mouse islets compared to the control islets. Exposure to butyrate did not affect IL-1β-induced changes in mRNA expression (Figure 4A–E).

**Figure 5:**
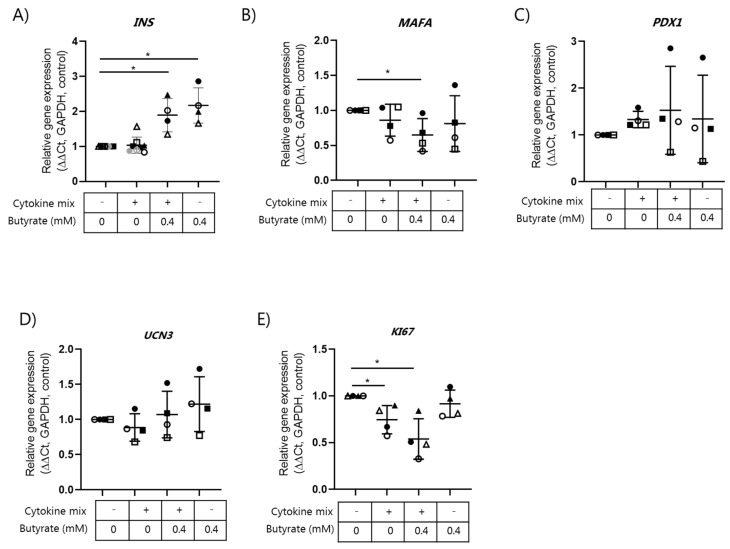
Expression of beta cell genes in EndoC-βH1 cells exposed to the cytokine mix and butyrate. Human EndoC-βH1 cells were exposed to a cytokine mix (500 pg/mL IL-1β + 2 ng/mL INF-γ + 2 ng/mL TNF-α) in the presence or absence of 0.4 mM butyrate for 6.5 days. Relative mRNA expression of (**A**) *INS*, (**B**) *MAFA*, (**C**) *PDX1*, (**D**) *UCN3*, and (**E**) *KI67* were analyzed using qPCR. Expression levels were normalized to the expression of *GADPH* (ΔCt) and control Endo C-βH1 cell expressions were set to 1 (ΔΔCt). Data are shown as means ± SD for *n* = 4 and analyzed by a 2-sided *t*-test. * *p* < 0.05 vs. control.

In EndoC-βH1 cells, no regulation of the selected beta cell genes by cytokines was observed when compared to non-exposed control cells (Figure 5A–E). However, butyrate significantly increased the expression of the *INS* gene (Figure 5A). In contrast, a significant reduction in *MAFA* mRNA in response to cytokines combined with butyrate was observed (Figure 5B). Expression of the proliferation marker *KI67* was significantly decreased by the cytokine mix alone or when it was combined with butyrate (Figure 5E).

### 2.5. Butyrate Affects IL-1β Regulated Gene Expression in Mouse Islets

**Figure 6:**
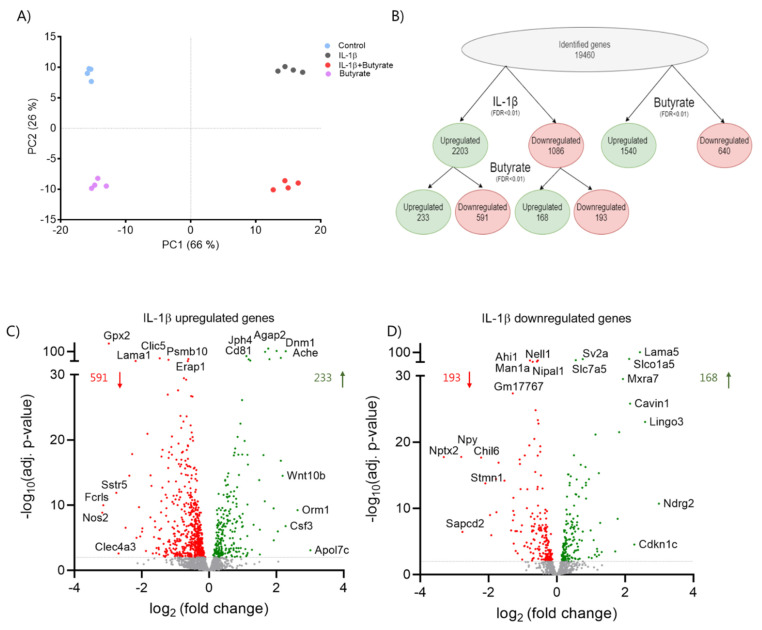
Butyrate and IL-1β affect global gene expression in mouse islets. Mouse islets were cultured for 10 days in the absence or presence of 50 pg/mL IL-1β or 0.2 mM butyrate. RNA was isolated for RNA-sequencing analysis. (**A**) Principle component analysis (PCA) plot of the top 1000 most variable genes showing the clustering of the samples by exposure. Each dot represents a sample. The first two principal components (PC) are shown, and the percentage of variance explained by each PC is indicated. (**B**) Overview of differentially expressed genes between control vs. IL-1β (FDR adjusted *p*-value < 0.01), non-exposed vs. butyrate (FDR adjusted *p*-value < 0.01), and IL-1β vs. IL-1β plus butyrate (FDR adjusted *p*-value < 0.01) exposed mouse islets. (**C**,**D**) Volcano plots showing the effect of butyrate on IL-1β up- (2203 genes) or down-regulated genes (1086 genes). Each dot represents an individual gene. Red dots represent significantly downregulated genes (FDR adjusted *p*-value < 0.01), Green dots represent significantly upregulated genes (FDR adjusted *p*-value < 0.01). The top 5 most significantly (IL-1β vs. IL-1β plus butyrate) and top 5 most regulated genes are labeled. Data are shown as means of *n* = 4 independent experiments.

**Figure 7:**
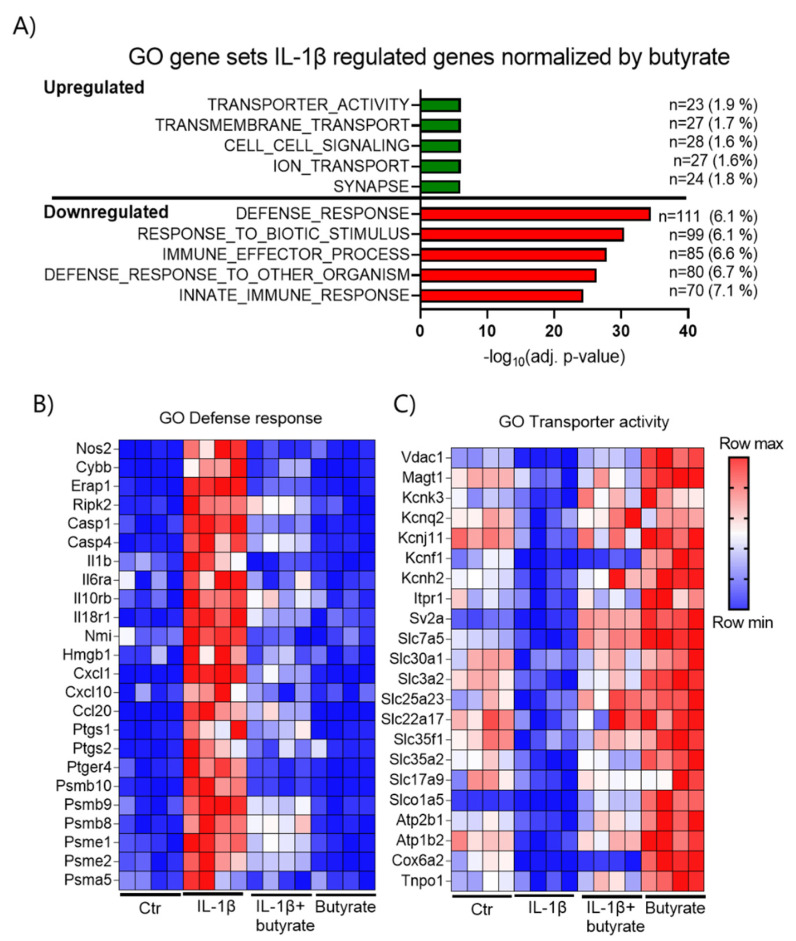
Analysis of genes regulated by IL-1β and normalized by butyrate in mouse islets. (**A**) Top 5 most significantly enriched GO terms of IL-1β-regulated genes normalized by butyrate; green: down-regulated by IL-1β and up-regulated by butyrate, red: up-regulated by IL-1β and down-regulated by butyrate. Bars show significance; the number (n) of butyrate regulated genes in the GO term, and the percentage of genes within the GO term are also shown. (**B**,**C**) Heatmaps displaying the relative gene expression levels of selected genes in GO term defence response, (**B**) and transporter activity (**C**). Blue = low expression. Red = high expression. Numerical values for A and the complete list of butyrate regulated genes found in the GO terms are provided in Supplementary Table S1.

In order to further characterize the effects of butyrate on IL-1β-induced dysfunction, we analyzed global gene expression in mouse islets by RNA-sequencing (RNA-seq). RNA was isolated from mouse islets exposed for 10 days to IL-1β (50 pg/mL) and/or butyrate (0.2 mM) and from the control islets (Figure 6). The expression profiles were clearly different between the four conditions of islet exposure as shown in the principal component analysis (PCA) plot, in which four clusters appeared from the four independent experiments (Figure 6A). First, we analyzed how butyrate affected IL-1β regulated gene expression. IL-1β significantly affected the expression of 3289 genes (17%), of which 2203 genes were upregulated and 1086 genes were downregulated (FDR < 0.01). Butyrate normalized the expression of 759 IL-1β regulated genes (23%) of which 591 genes were upregulated and 168 genes were downregulated (Figure 6B). Among the IL-1β upregulated genes, those most downregulated by butyrate were *Nos2*, *Gpx2*, and *Sstr5* (Figure 6C). *Ndrg2*, *Lingo3*, and *Lama5* were some of the IL-1β downregulated genes most upregulated by butyrate (Figure 6D). In agreement with the qPCR results, butyrate had no effect on beta cell maturity genes, which were downregulated by IL-1β (data not shown). To further investigate the characteristics of the IL-1β regulated genes normalized by butyrate, we performed a gene ontology (GO) analysis. Interestingly, butyrate significantly downregulated genes enriched in inflammatory pathways that were induced by IL-1β, while the GO analysis revealed only the smaller effects of butyrate on IL-1β downregulated genes (Figure 7A). Butyrate downregulated IL-1β upregulated pro-inflammatory genes encoding cytokines, chemokines, enzymes producing pro-inflammatory mediators *Nos2* (iNOS), *Ptgs1/2* (Cox1/2), *Cybb* (Nox2), the immunoproteasome subunits *Psmb8* (β5i), *Psmb9* (β1i), *Psmb10* (β2i), *Psme1* (PA28α), and *Psme2* (PA28β) (Figure 7B, Supplementary Figure S4 and Table S1). In contrast, IL-1β upregulated genes normalized by butyrate were associated with secretion/transport, among other ion transporters such as potassium transporters *Kcnk3*, *Kcnq2*, *Kcnj11*, *Kcnf1*, *Kcnh2* (Figure 7A,C, Supplementary Table S1). Compared to non-exposed islets, butyrate alone also upregulated several of the transport-associated genes (Figure 7C). In general, many of the IL-1β regulated genes normalized by butyrate were also regulated by butyrate alone (Supplementary Figure S5).

### 2.6. Butyrate Inhibits Cell Cycle Related Genes and Upregulates Transport-Associated Genes

**Figure 8:**
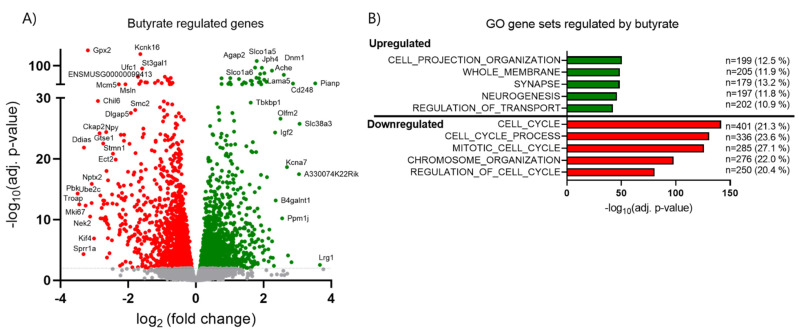
Butyrate alone affects the gene expression in mouse islets. (**A**) Volcano plot showing the effect of 0.2 mM butyrate alone vs. control gene expression in mouse islets exposed for 10 days. Each dot represents an individual gene. Red dots represent significantly downregulated genes (2181) (FDR adjusted *p*-value < 0.01). Green dots represent significantly upregulated genes (1540) (FDR adjusted *p*-value < 0.01). Selected genes are labeled. (**B**) Top 5 most significantly enriched GO terms are shown for both the up- (green) and down- (red) regulated genes by butyrate vs. control. Bars show significance; the number (n) of butyrate regulated genes in the GO term and percentage of genes within the GO term are also indicated. Numerical values for (B) and the complete list of butyrate regulated genes found in the GO terms are provided in Supplementary Table S2.

Since we observed significant effects of butyrate on beta cell proliferation and insulin secretion, we investigated which pathways were affected by butyrate alone compared to control islets by GO analysis (Figure 7B,C and Figure 8A,B). A total of 3721 genes (19.1%) were significantly (FDR < 0.01) regulated (1540 up and 2181 down) by butyrate (Figure 6B). GO analysis showed that upregulated genes were related to the pathways involved in cell projection, organization, neurons, and transport, whereas downregulated genes showed involvement in cell cycle regulation and chromosome organization (Figure 8B, Supplementary Table S2). In total, 55 out of 123 genes in the cell cycle KEGG pathway were reduced by butyrate (Supplementary Figure S6). These results support our findings that butyrate inhibits beta cell proliferation by regulating key cell cycle genes (Figure 3).

Together, the RNA-seq results suggest that butyrate has an anti-inflammatory role in IL-1β-induced dysfunction and normalizes or further upregulates the expression of secretion/transport-associated genes, while cell cycle related genes are inhibited.

## 3. Discussion

In this study, we investigated the effect of the SCFA, butyrate, on beta cell function in presence and absence of inflammatory stress induced by long-term exposure to non-cytotoxic concentrations of cytokines, which resembles the slow onset of inflammation in T2D [12]. We showed that butyrate protected beta cells from cytokine-induced impairment of GSIS and reduced insulin content in three different beta cell models; isolated mouse islets, human EndoC-βH1 beta cells and rat insulinoma INS-1E beta cells. The effects of butyrate were associated with changes in pro-inflammatory and secretion/transport-related gene expression.

In line with our previous study [12] and other studies [6], exposure of beta cells to pro-inflammatory cytokines induced a state of dedifferentiation. This was characterized by impaired insulin secretion, reduced proliferation and decreased expression of beta cell specific genes such as *Ins1*, *Ins2*, and *MafA* in mouse islets. Surprisingly, none of the beta cell genes *INS*, *MAFA*, *PDX1*, or *UCN3* were affected in EndoC-βH1 cells exposed to the non-cytotoxic cytokine mix for 7 days. However, *IAPP* (data not shown), which is known to associate with impaired insulin secretion and reduced functionality of the pancreatic islets in T2D patients [30], was significantly increased. These data, combined with GSIS data, indicate that the cytokine mix dose used in the EndoC-βH1 cells was sufficient to induce dysfunctional beta cells. In our previous study, we showed that the reduced expression of beta cell-specific genes correlated with histone modifications, mostly acetylation, near the transcription start site of the affected genes, and the function was restored after removal of the cytokines [12]. This indicates that the changes are reversible, which has also been demonstrated by others [6,31]. As butyrate is known to act as a histone deacetylase (HDAC) inhibitor [32], and HDAC inhibitors positively influence beta cell function [33,34,35,36], we hypothesized that the protective role of butyrate could be to modify the chromatin environment around beta cell specific genes through HDAC inhibition. However, in mouse islets, butyrate did not reverse the IL-1β inhibited expression of genes such as *Ins1*, *Ins2*, *Ucn3*, *MafA*, *Glut2* (*Slc2a2*), *Gck*, or other beta cell-specific transcription factors, suggesting that other genes or pathways may explain the positive effects by butyrate on beta cell function.

In contrast, butyrate downregulated several IL-1β-induced inflammatory genes encoding chemokines, interleukins, immunoproteasome subunits, and enzymes producing inflammatory mediators. Other studies have also reported on the anti-inflammatory properties of butyrate, which are thought to be mediated through inhibition of NFκB [37,38,39,40]. In vivo downregulation of chemokines such as *Cxcl1* and *Cxcl10* may be important to reduce the recruitment and activation of immune cells, thereby reducing islet inflammation and improving beta cell function [41]. One of the most downregulated genes was nitric oxide synthase 2 (*Nos2*), encoding the iNOS enzyme. iNOS is responsible for nitric oxide formation, which has been shown to impair beta cell function and affect the expression of multiple genes, leading to detrimental effects [42,43]. The positive effect of butyrate on many IL-1β upregulated genes could therefore be secondary to the inhibition of *Nos2* rather than direct effects of butyrate.

Moreover, the generation of prostaglandins and reactive oxygen species induced by IL-1β are likely inhibited by butyrate, as the gene expression of cyclooxygenase-2 (*Cox-2*) and NADPH oxidase 2 (*Nox2*), respectively, were normalized by butyrate. As these enzymes dramatically reduce beta cell function and affect the redox state [44,45], the protective role of butyrate may be through the reduction of oxidative and nitrosative stresses, in agreement with a recent study [18]. Potentially, butyrate could also upregulate antioxidant enzymes, as has been demonstrated by others [46,47], but this was not observed and in fact the most downregulated gene by butyrate in the absence of IL-1β was glutathione peroxidase 2 (*Gpx2*). Other members of the GPX family and members of the superoxide dismutase *(SOD)* and peroxiredoxin (PRDX) families and catalase (*Cat*) were also downregulated by butyrate.

In addition, butyrate also upregulated several genes both in presence and absence of IL-1β. Many of these were transporters or secretion-related genes present in neuronal related GO-terms such as neurogenesis and synapse. This could indicate that multiple genes are commonly expressed in both beta cells and neurons and function similarly to store and release hormones/neurotransmitters in response to stimuli [48]. The upregulation of some of these genes may play a role in the regulation of insulin secretion e.g., by regulation of the docking and fusion of the insulin granules to the plasma membrane [49]. For example *Stx1a* and *Stx1b*, encoding syntaxin-1 and *Snap23* but not *Snap25*, which are all part of the SNARE complex, were upregulated by butyrate, several Ca2+ sensing synaptotagmins (*Syt3*, *Esyt3*, *Syt7*, *Syt12*), and *Casp2*, which together regulate insulin granule priming, exocytosis, and stability [50,51]. One of the most regulated genes by butyrate alone, with a fold change of 8, was the glutamine transporter *slc38a3*/SN1, which is also expressed in neurons [52]. Previous studies have shown that glutamine and the subsequent formation of glutamate may modulate insulin secretion in multiple ways [53].

The fact that several of the secretion-related genes were also upregulated by butyrate alone indicates that the action of butyrate is not only through the counteraction of IL-1 signaling. This is supported by the observation that butyrate on its own enhanced GSIS in mouse islets and EndoC-βH1 cells. In agreement with this, a recent study showed that the HDAC inhibitor MS-275 reprogrammed islet gene expression and potentiated insulin secretion via the upregulation of secretion-related genes such as genes involved in Ca2+ and cAMP signaling. Half of the genes were also regulated by butyrate (at 5 mM), supporting the role of butyrate as a HDAC inhibitor [54]. In INS-1E cells, butyrate alone did not potentiate GSIS, indicating model-specific effects.

In addition to the positive effect of butyrate on insulin secretion, we observed a strong inhibition of cell proliferation as measured by a decrease in EdU positive cells and inhibition of cell cycle-related genes. This is in line with studies showing that butyrate has anti-cancer activity [32,55] and a study in the rat insulinoma (RIN) cell line [56], but in contrast to studies in diabetic rodents where butyrate or a HDAC3 inhibitor increased beta cell proliferation [27,57]. This discrepancy could be due to other factors, which regulate proliferation in vivo or differences in the concentration used or the metabolic state of the cell. In some cells, butyrate may be metabolized and promote cell proliferation by acting as an energy source, providing AcetylCoA to HATs to enhance their activity, while in other cells butyrate will be metabolized slower, the intracellular levels will be higher, and butyrate may function as a HDAC inhibitor [58]. How this strong inhibition of cell proliferation relates to function remains in question, but proliferation is in general described to be associated with decreased functionality and beta cell immaturity [59].

In addition to its action as an HDAC inhibitor, butyrate may also mediate its function by binding to the GPCRs FFAR2 and FFAR3 to activate downstream signaling. Recent studies have shown that FFAR2 and FFAR3 are expressed in beta cells [18,60], receptor deficiency models, and pharmacological and SCFA treatments have shown that activation of these receptors both stimulates and inhibits insulin secretion, but the results are not consistent [61]. It is likely that time of exposure and concentration of butyrate determine its mechanism of action. Acute exposure of beta cells to SCFAs may result in immediate signaling events by GPCR activation, whereas long-term exposures likely result in changes in gene expression by inhibition of HDAC. This could be one of the reasons why Lorza-Gil et al. [19] did not see an effect of butyrate on GSIS in human pseudo islets and mouse islets and even an inhibition in INS-1E cells, which is in contrast to our findings. Through the inhibition of HDAC, butyrate may promote the acetylation of non-histone proteins and histones that may affect the chromatin structure and accessibility of genes, ultimately affecting gene expression [62].

Taken together, our results show that butyrate improves beta cell function and can prevent cytokine-induced GSIS impairment. These effects correlated with the inhibition of inflammatory genes and the upregulation of secretion-related genes. However, the precise mechanisms by which butyrate regulates gene expression, the importance of specific changes, and whether the regulation is direct or indirect warrant further studies. Furthermore, future studies are needed in vivo to reveal the functions of butyrate in a setting with a complex exposure of islet cells to different cytokines, high glucose, and free fatty acids, as in T2D individuals. Since butyrate protected against cytokine-induced dysfunction in both mouse islets and two different beta cell lines, and considering the strong association between butyrate producing gut bacteria, glucose metabolism, and beta cell function in large human studies [24], we suggest that butyrate will be relevant to study for its potential for therapeutic use in prevention or treatment of T2D.

## 4. Materials and Methods

### 4.1. Mouse Islet Isolation and Beta Cell Line Culture

Pancreatic islets from 12 week-old male C57BL/6NRj mice (Janvier, Saint Berthewin Cedex, France) were isolated by perfusion via the bile duct of the pancreas with Liberase (Roche, Hvidovre, Denmark) as previously described [63]. Islets were isolated from 6–8 mice with an approximate yield off 200–250 islets per mouse. For GSIS, apoptosis and proliferation analysis, islets from individual mice were used. For RNA isolation, pools of islets from several mice were used.

After isolation, mouse islets were cultured for one day in RPMI 1640 medium with GlutaMAX (Gibco, Life Technologies, Roskilde, Denmark) supplemented with 10% fetal bovine serum (FBS; Biosera, Herlev, Denmark) and 1% penicillin/streptomycin (100 U/mL penicillin, 100 μg/mL streptomycin) (P/S; Gibco, Life Technologies). Thereafter islets were cultured for 10 days in RPMI 1640 supplemented with 2% human serum (HS; BioWhittaker, Lonza, Basel, Switzerland) and 1% P/S, with culture media being changed after 5 days. Experiments involving animals were approved by the local ethics committee, and animals were housed according to the Principles of Laboratory Care.

The INS-1E rat insulinoma cell line, a kind gift from Claes Wollheim (University of Geneva, Switzerland), was maintained in RPMI 1640 medium supplemented with 10% heat-inactivated FBS, 1% P/S and 50 μM β-mercaptoethanol (Life Technologies). The cells were seeded at day 0, followed by treatment at day 2, then cultured for further 3 days. Human EndoC-βH1 cells were cultured in serum-free DMEM (Life Technologies) containing 6.5 mM glucose, as previously described [64]. The cells were seeded at day 0 followed by treatment at day 1 and re-treatment/media change at day 4. Total culture period was 7 days.

Mouse islets and INS-1E cells were exposed to recombinant mouse IL-1β (cat. 554577, BD Biosciences, Lyngby, Denmark) and/or sodium butyrate (B5887, Sigma, Soeborg, Denmark). Since human beta cells require several cytokines to induce dysfunction [11] we used IL-1β in combination with IFN-γ and TNF-α (all R&D Systems, Life Technologies) and/or sodium butyrate in EndoC-βH1 cells.

### 4.2. Glucose-Stimulated Insulin Release and Insulin Content

GSIS was performed with 25 islets per condition. First, islets were pre-cultured in Krebs–Ringer HEPES (KRBH) buffer with 2 mM D-glucose. This was followed by 30 min incubation in new KRBH buffer with 2 mM D-glucose and then 30 min of incubation in KRBH buffer with 20 mM D-glucose. After each incubation step, the buffer was collected and insulin was determined using an in-house ELISA. Cellular insulin and DNA content were analyzed in homogenates obtained after sonication of islets. Insulin secretion was normalized to DNA content, then analyzed by the Quant-IT PicoGreen dsDNA Reagent and Kit (Life Technologies).

INS-1E cells (6.5 × 10^4^ per well) were seeded in 24-well plates and exposed to IL-1β or butyrate alone, in combination, or left non-exposed. GSIS was performed as described above.

EndoC-βH1 cells (6.5 × 10^4^ per well) were seeded in 96-well plates and exposed to cytokines, butyrate, or left non-exposed, as described above. Prior to the GSIS assay 6.5 days post seeding, the cells were cultured overnight in media containing 2.8 mM glucose followed by 1-h starvation in KRBH buffer containing 0.5 mM glucose. Acute stimulation with 0.5 mM and 11.2 mM glucose with and without the addition of 50 µM forskolin in KRBH was subsequently performed for 45 min at day 7. Insulin secretion was normalized to the number of cells per well, counted at the end of experiment. Results were corrected for cell number as cells post assay were fixed and counted.

### 4.3. Cell Apoptosis Assay

Apoptotic cell death was measured in 25 mouse islets and EndoC-βH1 cells (5.5 × 10^4^ cells per well) in 96-well plates by the detection of DNA histone complexes released from the nucleus to the cytosol using Cell Death Detection ELISA PLUS (Roche), as described by the manufacturer. Apoptosis in mouse islets were corrected for DNA content using a Quant-IT PicoGreen dsDNA Reagent and Kit (Life Technologies) and EndoC-βH1 cell results were normalized to total cell number per 96-well plate.

### 4.4. Caspase 3/7 Activity

Analysis of caspase 3/7 activity was performed using the IncuCyte Live Cell Analysis System (Sartorius, Glostrup, Denmark), as described by manufacturer. EndoC-βH1 cells (5.5 × 10^4^ cells per well) were seeded in a 96-well plate and IncuCyte Caspase-3/7 Reagent (Sartorius) was added to the culture media to evaluate cells undergoing caspase 3/7 mediated apoptosis induced by the applied cytokine combinations. Data collection occurred every 12 h and pictures were captured with magnification 10x. Data were normalized to total cell number per 96-well plate.

### 4.5. Proliferation

Beta cell proliferation was analyzed in mouse islets cultured on laminin (Life Technologies) coated slides. EdU (5-ethynyl-2′-deoxyuridine) (Life Technologies) was added 24 h prior to fixation in 1% PFA and proliferating beta cells were detected by EdU incorporation using a Click-iT EdU Proliferation Assay (Life Technologies). Beta cells were identified by staining for pancreatic duodenal homeobox 1 (goat-anti PDX-1 1:5000, ab2027, BCBC) and DNA (Hoescht 1:2000, 33342, Life Technologies). Islet cells positive for both Pdx1 and EdU were counted as proliferating beta cells. Whole islets were examined by capturing z-stack images using a Zeiss Axio Observer with a spinning disk (Birkerød, Denmark). Zen software (Zeiss) was used for the quantification of the total number of Pdx1 positive cells positive for EdU. A total of 10 islets from three independent experiments were analyzed.

EndoC-βH1 cell proliferation was analyzed in 96-well plates (5.5 × 10^4^ cells per well). After 6.5 days of treatment, the cells were incubated with EdU for 1 h and fixed in 1% PFA. EdU incorporation was detected using the Click-iT, as described above. Cells were analyzed by fluorescent microscope (Olympus IX71, Ballerup, Denmark) and ImageXpress Micro Confocal Imaging System by acquiring 9 images per 96-well plate with magnification of 10×. MetaXpress software (Moleculare devices, San Jose, CA, USA) was used for quantification of cells positive for EdU. Approximately 50,000 cells per well were examined.

### 4.6. Gene Expression Analysis

A pool of 300 islets per condition were, after the culture period, lysed in Trizol and RNA was extracted using a Direct-zol RNA-mini prep kit according to the manufacturer’s protocol (Zymo Research, Nordic Biosite, Copenhagen, Denmark). cDNA was synthesized using the qScript cDNA Super mix kit (Quanta Biosciences). Quantitative expression levels of mRNAs of interest were evaluated using TaqMan Gene Expression probes and performed on an ABI PRISM 7900HT Sequence Detection System (Applied Biosystems). Each sample was determined in triplicate and expression was normalized to the internal control, *Ppia*.

EndoC-βH1 cells were seeded at 2.3 × 10^6^ cells per well in 6-well plates. Cells were lysed in RLT-buffer (RNeasy Mini Kit, Qiagen, Copenhagen, Denmark) and RNA extraction was performed according to the manufacturer’s procedure (RNeasy Plus Mini Kit, Qiagen). Reverse transcription of RNA to cDNA was performed using the iScript cDNA Synthesis Kit (Bio-Rad, Copenhagen, Denmark). Real time qPCR was performed using TaqMan primer-probes (Primer design & Applied Biosystems, Life Technologies) on a ViiA7 Real-Time PCR System (Applied Biosystems). Each sample was determined in triplicate, and expression was normalized to the internal control, *GAPDH*.

### 4.7. RNA Sequencing

RNA was isolated from 500 mouse islets using the RNeasy^®^ Micro Kit (Qiagen) and biological replicates were sequenced per experimental condition (*n* = 4). Single-end sequencing libraries were constructed from 250 ng of isolated RNA using the NEBNext^®^ Ultra™ II RNA Library Prep Kit #E7770S (Illumina^®^, San Diego, CA, USA) and sequencing was performed on a HiSeq 4000 System (Illumina^®^). Quality metrics were assessed by FastQC [65] before the reads were mapped to the GRCm38 (mm10) genome using STAR v2.5.3a [66]. Differential gene expression was determined using the R package DESeq2 with false discovery rate (FDR) *p*-values adjusted by the Benjamini–Hochberg method [67]. Differential expression was defined as FDR-adjusted *p*-values < 0.01. To identify the functional categories of differential expressed genes, gene ontology (GO) analysis was performed using the MSigDB database v7.2 (Molecular Signature Databases, https://www.gsea-msigdb.org/gsea/msigdb/, accessed on 22 December 2020) [68]. Due to an upper limit of genes to be analyzed in the analysis, 80 genes downregulated by butyrate alone were excluded from the analysis. KEGG pathway analysis was performed using ShinyGOV0.61 (ShinyGO v0.61, http://bioinformatics.sdstate.edu/go/, accessed on 5 January 2021) [69]. Tbe RNA sequencing (RNA-seq) datasets generated for this study are available at GEO NCBI, accession GSE180317.

### 4.8. Statistical Analysis

Results are shown as means ± SD. Comparison vs. the respective control group was made by Student’s paired two-tailed *t*-test or Mann–Whitney test. Statistical analysis of GSIS data was performed on log10-transformed data due to variance. *p*-values less than 0.05 were considered statistically significant.

## Data Availability

RNA-sequencing (RNA-seq) datasets for this study are available at GEO NCBI, accession GSE180317.

## Supplementary Materials

The following are available online at https://www.mdpi.com/article/10.3390/ijms221910427/s1.

## Abbreviations

T2D	Type 2 Diabetes
SCFAs	short chain fatty acids
HDAC	histone deacetylase
GSIS	glucose-stimulated insulin secretion
GPCR	G-protein coupled receptors
PCA	principal component analysis
GO	gene ontology
