# Supplementary material for: Butyrate Protects Pancreatic Beta Cells from Cytokine-Induced Dysfunction
Source: Int J Mol Sci. 2021 Sep 27;22(19):10427. doi: 10.3390/ijms221910427 (PMC8508700; doi:10.3390/ijms221910427)
Supplement: Supplementary file 1 [file ijms-22-10427-s001.zip › ijms-1393654-supplementary.pdf]

Supplementary Figure 1

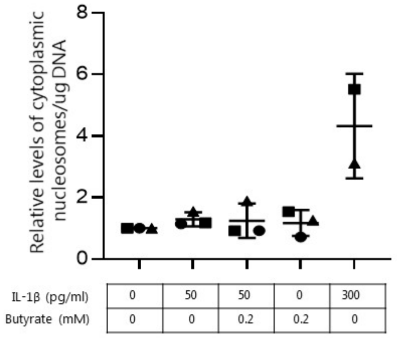

Supplementary Figure 1: IL-1b and butyrate effects on apoptosis in mouse islets.
Apoptosis was measured by analysis of cytoplasmic nucleosome levels in lysates from mouse islets exposed to 50 pg/ ml IL-1β and/or 0.2 mM butyrate or left non-exposed for 10 days. Two day stimulation with 300 pg/ml IL-1β served as positive control for induction of apoptosis, Data are shown as as means ±SD for n=2-3.

Supplementary Figure 2

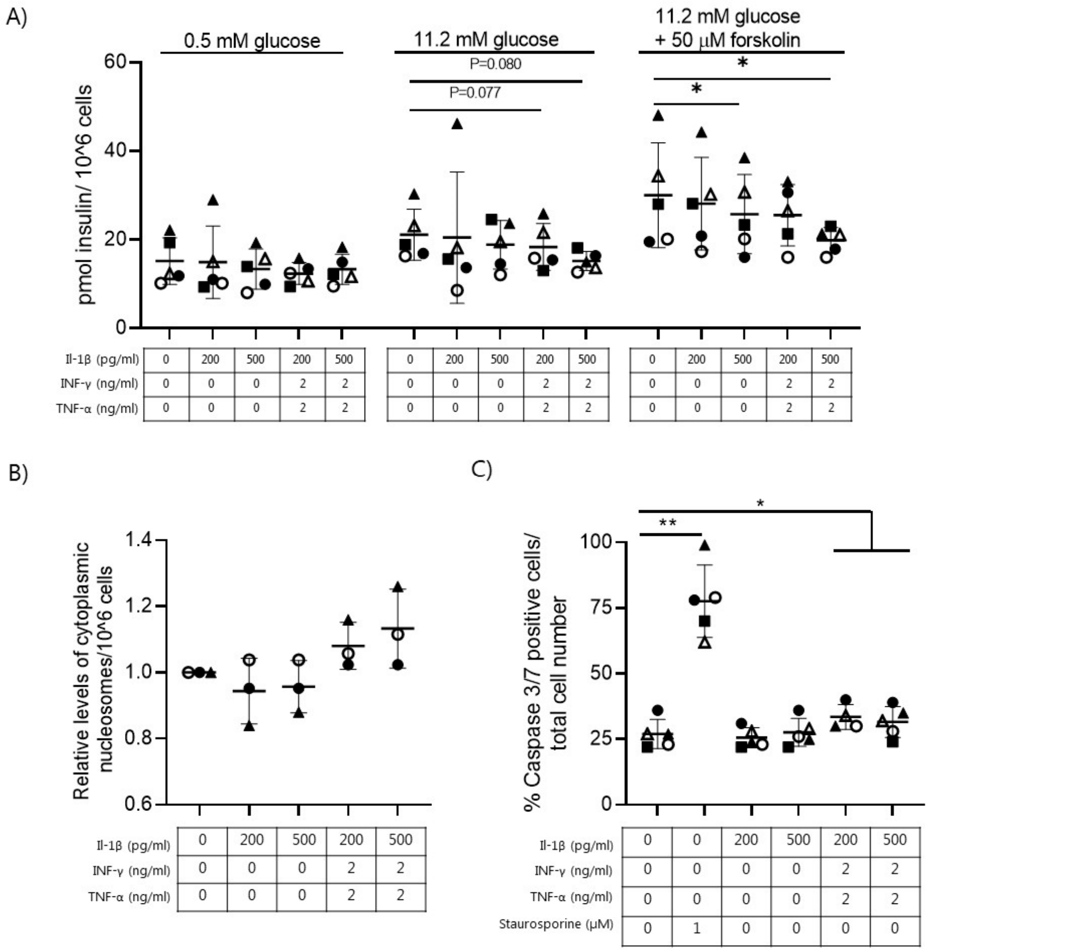

Supplementary Figure 2: Effect of cytokines on beta cell dysfunction and apoptosis in human EndoC-βH1 cells.

A) Human EndoC-βH1 cells were cultured for 7 days in the absence or presence of indicated concentrations of IL-1β combined with IFN-γ and TNF-α to determine the combination of cytokines sufficient to induce beta cell dysfunction. Insulin secretion was measured by static batch incubations for 45 min in response to 0.5 mmol/l glucose, 11.2 mmol/l glucose and 11.2 mmol/l glucose + 50µM forskolin and normalized to cell number. Each condition is tested in quadruplicates. Data are shown as means ±SD for n=5 and analyzed by a 2-sided paired t-test. *p<0.05 vs control.

Cytokine induced apoptosis in EndoC-βH1 cells exposed to the indicated combinations of cytokines was evaluated by B) cytoplasmic nucleosome level in lysates (n=3)or by C) caspase 3/7 activation (n=5). 1 µM Staurosporine was used as a positive control for induction of apoptosis . Each condition was tested in biological triplicates. Data are normalized to total cell number/well and shown as means ± SD. Data was analyzed by 2-sided paired t-test *<p<0.05, **p<0.01 vs control.

Supplementary Figure 3

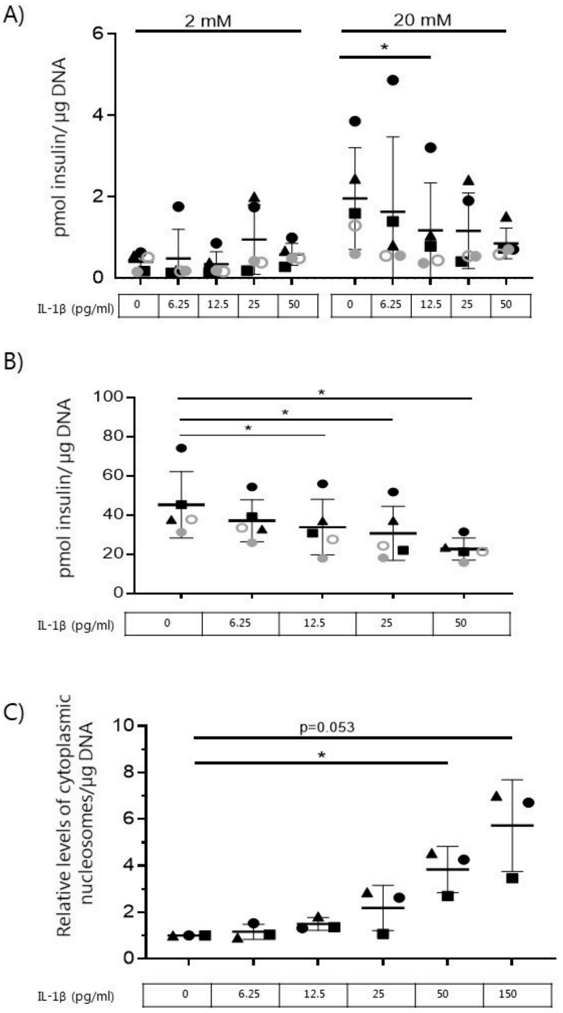

Supplementary Figure 3: Dose-dependent effect of IL-1β on beta cell dysfunction and apoptosis in rat INS-1E cells.
INS-1E cells were exposed to increasing concentration of IL-1β (0-50 ng/ml) for 72 hrs. A) Insulin secretion was measured by static batch incubations for 30 min in response to 2 mmol/l glucose followed by 20 mmol/l and normalized to DNA content. B) Total insulin content was measured post-glucose stimulation (n=5). C) Apoptosis was determined in lysates from INS-1E cells after 3 days exposure to increasing concentrations of IL-1β (0-50 ng/ml). As a positive control of apoptosis INS-1E cells were exposed to 150 pg/ml IL-1β for 24h (n=3). Data was normalized to DNA content of cells. Data are shown as means ±SD and analyzed by 2-sided paired student t-test *$p<0.05$.

Supplementary Figure 4

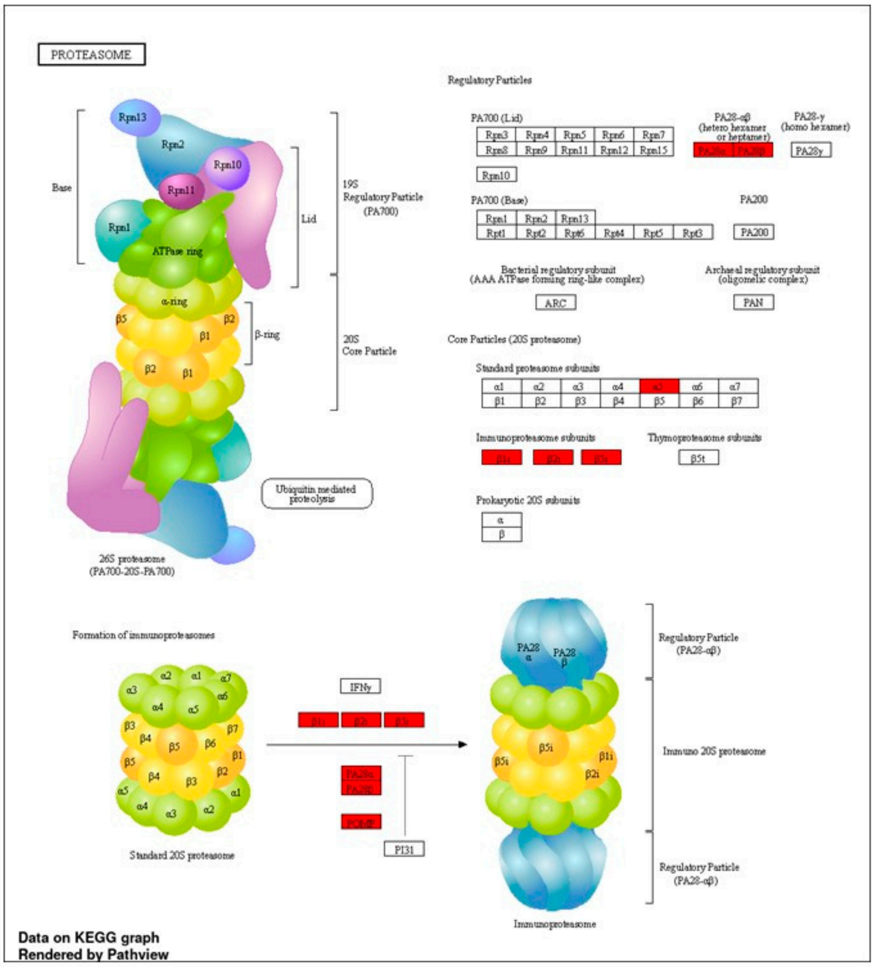

Supplementary Figure 4: IL-1β regulated genes downregulated by butyrate in the Proteasome KEGG pathway.
Overview of the genes in the Proteasome KEGG pathway. The genes downregulated by butyrate are indicated in the red boxes. Pathway map generated by using the ShinyGO v0.61 webtool.

Supplementary Figure 5

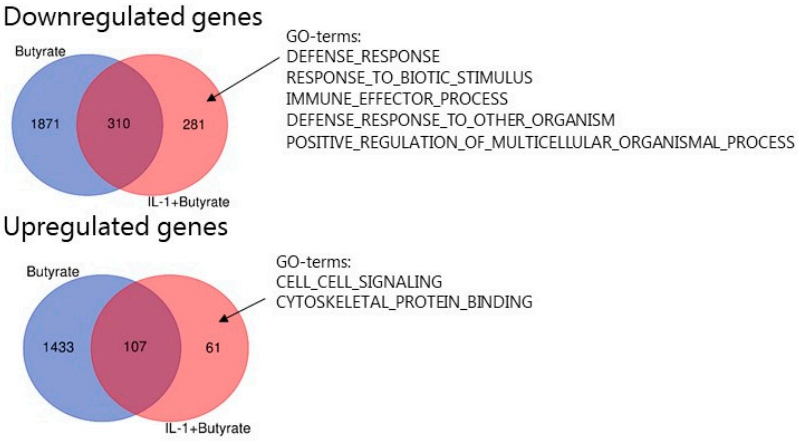

Supplementary Figure 5: Genes regulated by butyrate in presence and absence of IL-1β.
Venn diagram showing the number of genes down- and upregulated by butyrate in presence or absence of IL-1β. Overlapping circles show genes that are both regulated by butyrate alone and in presence of IL-1β. GO-terms significantly regulated by butyrate in the presence of IL-1β are indicated.

Supplementary Figure 6

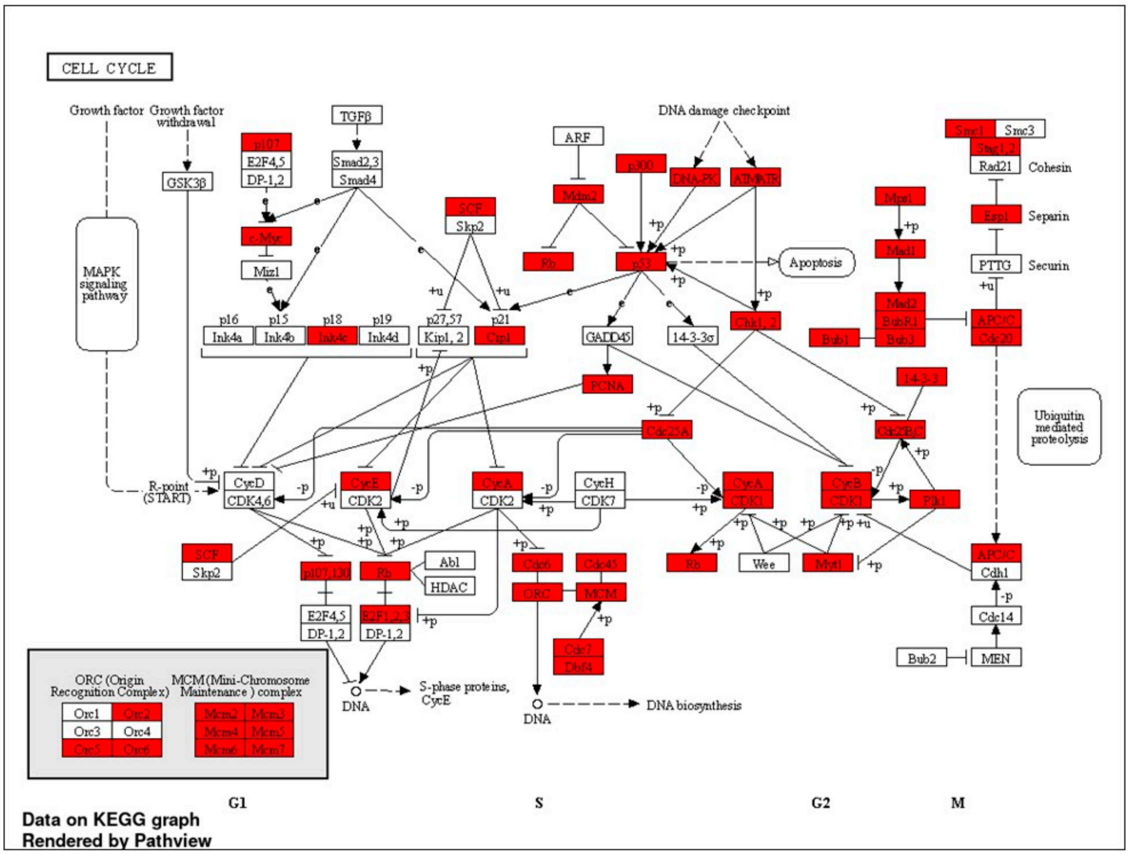

Supplementary Figure 6: Genes downregulated by butyrate in the cell cylcle KEGG pathway.
Overview of the genes in the cell cycle KEGG pathway. The genes downregulated by butyrate are indicated in the red boxes.
Pathway map generated by using the ShinyGO v0.61 webtool.

KEGG pathway enriched for butyrate downregulated genes

Functional Category	Enrichment FDR	Genes in list	Total genes
Cell cycle	1.2E-21	55	123
DNA replication	6.3E-09	19	35
Metabolic pathways	1.0E-07	192	1301
Oocyte meiosis	1.2E-07	34	115
P53 signaling pathway	6.4E-06	23	71
Human T-cell leukemia virus 1 infection	1.3E-04	46	235
Homologous recombination	1.3E-04	15	41
Fanconi anemia pathway	5.3E-04	16	51
Thyroid hormone signaling pathway	1.0E-03	26	115
Progesterone-mediated oocyte maturation	1.0E-03	22	90

Supplementary Table S1: Over-represented gene ontology (GO) terms for IL-1β regulated genes normalized by butyrate

GO term<br>Downregulated genes by butyrate	Butyrate regulated genes in GO term	Genes in GO term	Adj. p-value
DEFENSE_RESPONSE	111	1814	3.60E-35
RESPONSE_TO_BIOTIC_STIMULUS	99	1615	3.61E-31
IMMUNE_EFFECTOR_PROCESS	85	1292	1.58E-28
DEFENSE_RESPONSE_TO_OTHER_ORGANISM	80	1203	4.47E-27
INNATE_IMMUNE_RESPONSE	70	986	4.42E-25
**Upregulated genes by butyrate**			
TRANSMEMBRANE_TRANSPORT	27	1608	9.08E-07
TRANSPORTER_ACTIVITY	23	1199	9.08E-07
CELL_CELL_SIGNALING	28	1809	9.08E-07
ION_TRANSPORT	27	1696	9.08E-07
SYNAPSE	24	1357	9.95E-07

Downregulated genes:

**Defense response**
NOS2, DDX58, FCER1G, RIPK2, TNFAIP3, TRIM5, RELB, SAMHD1, RNF135, APOBEC3F, B2M, SLC11A1, BST2, TYROBP, PSMA5, TREM2, IRF7, PARP9, IFNAR2, HMGB1, SERPING1, DDX60, DTX3L, SERPINB9, STX4, IGHM, C1QC, C1QB, TLR2, TP53, PRDX1, IFIH1, IFI27, CYBB, GSDMD, RNASET2, UNC93B1, ZC3HAV1, IL1B, CXCL10, MOV10, LYZ, IL10RB, APOBEC1, RTP4, TNFRSF1B, PTGER4, PRDX2, CD68, PSME2, NMI, APOE, MUC13, PSME1, PSMB10, PSMB8, PSMB9, IRGM, PARP14, PAK3, ERAP1, CLEC4A, TRAFD1, CCL20, CASP1, CLDN1, ASS1, GBP2, GBP4, ARID5A, NR1H4, CASP4, PLD4, LY86, TYK2, AQP4, GBP6, GBP7, NAIP, MPEG1, STYK1, IL6R, STAB1, LGALS8, TNIP2, PTGS2, CXCR4, CXCL1, APOA2, ITGB2, IL18R1, CST3, IGF1, PLA2G7, ITGB1, GAL, ADCY8, KLF4, ISL1, PLD3, C2CD4A, PTGS1, IL17RE, BDNF, ADCY1, BMPR1B, BACE2, SCN9A, LXN, LIPA, PARP4
**Response to biotic stimuli**
NOS2, DDX58, FCER1G, RIPK2, TNFAIP3, TRIM5, RELB, SAMHD1, RNF135, APOBEC3F, B2M, SLC11A1, BST2, TYROBP, PSMA5, TREM2, IRF7, PARP9, IFNAR2, HMGB1, SERPING1, DDX60, DTX3L, SERPINB9, STX4, IGHM, C1QC, C1QB, TLR2, TP53, PRDX1, IFIH1, IFI27, CYBB, GSDMD, RNASET2, UNC93B1, ZC3HAV1, IL1B, CXCL10, MOV10, LYZ, IL10RB, APOBEC1, RTP4, TNFRSF1B, PTGER4, PRDX2, CD68, PSME2, NMI, APOE, MUC13, PSME1, PSMB10, PSMB8, PSMB9, IRGM, PARP14, PAK3, ERAP1, CLEC4A, TRAFD1, CCL20, CASP1, CLDN1, ASS1, GBP2, GBP4, ARID5A, NR1H4, CASP4, PLD4, LY86, TYK2, AQP4, GBP6, GBP7, NAIP, MPEG1, STYK1, IL6R, STAB1, LGALS8, TNIP2, PTGS2, CXCR4, CXCL1, CASP3, HPGD, GGT7, ABCC2, NFKB2, NFKBIB, PIM2, IVNS1ABP, PLAAT3, HERC6, BTBD17
**Immune effector process**
NOS2, DDX58, FCER1G, RIPK2, TNFAIP3, TRIM5, RELB, SAMHD1, RNF135, APOBEC3F, B2M, SLC11A1, BST2, TYROBP, PSMA5, TREM2, IRF7, PARP9, IFNAR2, HMGB1, SERPING1, DDX60, DTX3L, SERPINB9, STX4, IGHM, C1QC, C1QB, TLR2, TP53, PRDX1, IFIH1, IFI27, CYBB, GSDMD, RNASET2, UNC93B1, ZC3HAV1, IL1B, CXCL10, MOV10, LYZ, IL10RB, APOBEC1, RTP4, TNFRSF1B, PTGER4, PRDX2, CD68, APOA2, ITGB2, IL18R1, CST3, ACTB, CPB2, GPI, INPP5D, CYFIP1, CD33, ARPC5, FCGR2A, JAG1, CPN1, ARPC3, CR1L, FABP5, CAT, CD53, GUSB, GALNS, RORC, NCSTN, CTSS, ARHGAP45, AGA, FUCA1, DERA, PRDX4, STBD1, METTL7A, CSTB, VCL, CPPED1, CRISPLD2, WDR1
**Defense response to other organisms**
NOS2, DDX58, FCER1G, RIPK2, TNFAIP3, TRIM5, RELB, SAMHD1, RNF135, APOBEC3F, B2M, SLC11A1, BST2, TYROBP, PSMA5, TREM2, IRF7, PARP9, IFNAR2, HMGB1, SERPING1, DDX60, DTX3L, SERPINB9, STX4, IGHM, C1QC, C1QB, TLR2, TP53, PRDX1, IFIH1, IFI27, CYBB, GSDMD, RNASET2, UNC93B1, ZC3HAV1, IL1B, CXCL10, MOV10, LYZ, IL10RB, APOBEC1, RTP4, PSME2, NMI, APOE, MUC13, PSME1, PSMB10, PSMB8, PSMB9, IRGM, PARP14, PAK3, ERAP1, CLEC4A, TRAFD1, CCL20, CASP1, CLDN1, ASS1, GBP2, GBP4, ARID5A, NR1H4, CASP4, PLD4, LY86, TYK2, AQP4, GBP6, GBP7, NAIP, MPEG1, STYK1, IL6R, STAB1, LGALS8
**Innate immune response**
NOS2, DDX58, FCER1G, RIPK2, TNFAIP3, TRIM5, RELB, SAMHD1, RNF135, APOBEC3F, B2M, SLC11A1, BST2, TYROBP, PSMA5, TREM2, IRF7, PARP9, IFNAR2, HMGB1, SERPING1, DDX60, DTX3L, SERPINB9, STX4, IGHM, C1QC, C1QB, TLR2, TP53, PRDX1, IFIH1, IFI27, CYBB, GSDMD, RNASET2, UNC93B1, ZC3HAV1, PSME2, NMI, APOE, MUC13, PSME1, PSMB10, PSMB8, PSMB9, IRGM, PARP14, PAK3, ERAP1, CLEC4A, TRAFD1, CCL20, CASP1, CLDN1, ASS1, GBP2, GBP4, ARID5A, NR1H4, CASP4, PLD4, LY86, TYK2, AQP4, GBP6, GBP7, NAIP, MPEG1, STYK1

Upregulated genes:

**Transmembrane Transport**
VDAC1, SLC30A1, KCNK3, KCNQ2, ITPR1, SV2A, KCNJ11, SLC7A11, ATP2B1, ATP1B2, MAGT1, SLC3A2, SLC7A5, KCNH2, SLC25A23, KCNF1, COX6A2, SLC22A17, SLC35A2, SLCO1A2, SLC17A9, SLC35F1, SHANK1, RASGRF1, DDIT3, PPARGC1A, PRKCI
**Transporter activity**
VDAC1, SLC30A1, KCNK3, KCNQ2, ITPR1, SV2A, KCNJ11, SLC7A11, ATP2B1, ATP1B2, MAGT1, SLC3A2, SLC7A5, KCNH2, SLC25A23, KCNF1, COX6A2, SLC22A17, SLC35A2, SLCO1A2, SLC17A9, SLC35F1, TNPO1
**Cell cell signaling**
VDAC1, SLC30A1, KCNK3, KCNQ2, ITPR1, SV2A, KCNJ11, SLC7A11, SHANK1, RASGRF1, DDIT3, PXK, BAIAP3ZDHHC2, RIMS3, AGRN, NTF4, ETV5, DLGAP3, CUX2, ADGRG1, HADH, ACVR1C, C1QTNF4, RASL10B, NDRG2, ADGRA2, CCDC88C
**Ion transport**
VDAC1, SLC30A1, KCNK3, KCNQ2, ITPR1, SV2A, KCNJ11, SLC7A11, ATP2B1, ATP1B2, MAGT1, SLC3A2, SLC7A5, KCNH2, SLC25A23, KCNF1, COX6A2, SLC22A17, SLC35A2, SLCO1A2, SHANK1, RASGRF1, DDIT3, PPARGC1A, PXK, MT3, EPB41
**Synapse**
VDAC1, SLC30A1, KCNK3, KCNQ2, ITPR1, SV2A, ATP2B1, ATP1B2, SHANK1, PRKCI, BAIAP3, ZDHHC2, RIMS3, AGRN, NTF4, ETV5, DLGAP3, MT3, EPB41, SEC22B, STX16, BCL11A, CPEB1, LAMA5

Supplementary Table S2: Over-represented gene ontology (GO) terms for butyrate regulated genes

**GO term**<br>**Downregulated genes by butyrate**	**Butyrate regulated genes in GO term**	**Genes in GO term**	**Adj. p-value**
GO_CELL_CYCLE	401	1881	1.79E-142
GO_CELL_CYCLE_PROCESS	336	1422	1.18E-131
GO_MITOTIC_CELL_CYCLE	285	1053	8.79E-127
GO_CHROMOSOME_ORGANIZATION	276	1253	7.01E-99
GO_REGULATION_OF_CELL_CYCLE	250	1226	2.07E-81
**Upregulated genes by butyrate**			
GO_CELL_PROJECTION_ORGANIZATION	199	1588	3.89E-51
GO_WHOLE_MEMBRANE	205	1721	1.55E-49
GO_SYNAPSE	179	1357	3.51E-49
GO_NEUROGENESIS	197	1674	7.15E-47
GO_REGULATION_OF_TRANSPORT	202	1588	5.60E-43

Downregulated genes:

**Cell cycle**
CDK1, CDC6, AURKB, NEK2, CENPF, PCNA, TP53, BRCA1, CHEK1, ATM, GEN1, CDC45, CCNE1, CCNE2, CCNB1, BUB1B, CDC20, RB1, FBXO5, PLK1, AURKA, CENPE, NDC80, SPDL1, MAD1L1, MAD2L1, KIF14, ESPL1, TACC3, JADE1, TTK, DDX3X, CDK5RAP2, DLGAP5, BLM, CDCA5, CDT1, EP300, PSME4, CUL4B, RPA2, NBN, RFWD3, HUS1, PRKDC, DUSP1, MYC, UBE2C, CDC23, BUB1, BUB3, RBL1, SIN3A, PCID2, BRD7, CCNA2, BRCA2, RAD51, TOP2A, NEK7, INCENP, NUMA1, PSRC1, NUSAP1, KNSTRN, SPAG5, KIF23, RACGAP1, PRC1, MCPH1, PPP1R10, DNA2, FANCD2, IL1B, NAA10, MKI67, USP16, ZWINT, MCM3, MCM2, POLA1, POLE, POLE4, POLA2, MCM6, MCM7, MCM5, MCM4, MCM8, PPM1D, NASP, PHF8, SMC1A, KIF22, KIF2C, SGO1, STAG2, STAG1, DSN1, CDCA8, KIFC1, KIF18B, KIF4A, KATNB1, KIF18A, HASPIN, RTEL1, DSCC1, NUF2, CENPW, CENPA, CENPC, NSL1, NCAPD3, NCAPD2, NCAPG, SMC4, SMC2, NCAPG2, NCAPH, CENPH, CENPK, CDC7, STOX1, DTL, TOPBP1, CCNF, PLK2, KNTC1, PLK4, CENPJ, HAUS4, CDC25B, TUBB, CDK2AP2, TPX2, DYNLL1, BRSK1, E2F1, GTSE1, CEP57, CEP70, CEP43, OFD1, CEP192, CEP135, CEP76, PCNT, CEP152, HMMR, CEP78, TUBB4B, APP, CCNB2, MASTL, TIPIN, RBBP8, ATAD5, PSMD14, CDKN1A, SLFN11, CLSPN, TAOK3, FOXM1, KLF4, E2F7, E2F8, ANLN, CDC25A, RRM2, PSMC3, PSME2, PSMF1, PSMA7, PSMD11, CUL1, FBXO7, PSME1, PSMA4, PSMD6, PSMB8, PSMB10, PSMD8, E2F6, MDM2, TRIM39, RPS27L, PIDD1, RNASEH2B, CNOT8, DBF4, PKMYT1, PLCB1, APPL1, DACT1, AVEN, CDKN2C, ECD, CKS2, CKS1B, CDK14, CCNG1, CCNG2, FBXL3, FBXL12, CCNI, GMNN, PIM2, EIF4EBP1, CUL7, KIF20B, KIF11, CCSAP, KIF20A, ECT2, CEP85, STIL, POC1A, DLG1, EME2, MDC1, INTS3, FBXW5, EGF, DRG1, CAV2, ZWILCH, MAP3K20, DDIAS, HRAS, ORC2, ORC6, MCM10, ORC5, TYMS, UBE2S, FBXL15, TAF2, MELK, GSPT1, PPAT, BCAT1, IQGAP3, DHFRP1, ID4, ZNF324, XRCC2, BIRC5, CKAP2, CEP55, JTB, STMN1, MAP4, XIAP, AAAS, TUBGCP3, TUBGCP2, TUBGCP5, WDR62, TBCE, EML1, LIG1, VCP, SPC25, SAPCD2, SNX9, RASA1, STAMBP, ANKLE2, PTPA, MYBL1, BRCC3, NPM1, PAXIP1, TAF1, DDX11, KAT2B, USP51, BRIP1, TRIM37, EPC1, CENPX, SLX4, POLD1, ESCO2, FANCM, MSH3, RFC2, RFC3, RAD54B, SMARCAD1, KNL1, CENPQ, TIMELESS, NEK10, DDRGK1, PCLAF, CALM2, KLHL21, NUBP1, CEP295, CEP120, ASPM, NSUN2, MAP2K1, GAS2, NUDT16, DACH1, BARD1, FIGNL1, SSTR5, CBX5, PPP2R5B, XPO1, GATA6, MLXIPL, NOTCH1, CDC123, PPP2R3B, TBRG1, RRAGB, IRF1, TP53INP1, CAB39L, INHA, WDR6, PRR11, HEPACAM2, NIN, C2CD3, SSX2IP, CNTLN, SASS6, FANCA, PSMC3IP, PHGDH, SIRT1, YEATS4, DUSP3, NUP214, SKA3, SKA1, TUBA1B, CLIP1, KIF15, TBCD, TUBB6, WDHD1, RRM1, PBK, H2AX, FBXO4, ATR, COPS5, ACTB, HCFC1, HELLS, MIS18A, SMARCB1, CHAF1A, CHAF1B, EXO1, UHRF1, CHTF18, ERCC6L, OIP5, MIS18BP1, SUV39H1, HJURP, SUV39H2, EID1, ZMYND11, PRKACB, THOC1, LIN9, TRIM21, CRY1, JUN, BIRC2, NR4A1, E2F2, CCNDBP1, LIN54, TRIOBP, POC5, FANCI, PPP1CA, ANAPC13, SPC24, CDCA2, USP39, SENP5, CDCA3, PIMREG, MAPK4, GAK, GSPT2, MNS1, SIAH1, MRPL41, RGS2
**Cell cycle process**
CDK1, CDC6, AURKB, NEK2, CENPF, PCNA, TP53, BRCA1, CHEK1, ATM, GEN1, CDC45, CCNE1, CCNE2, CCNB1, BUB1B, CDC20, RB1, FBXO5, PLK1, AURKA, CENPE, NDC80, SPDL1, MAD1L1, MAD2L1, KIF14, ESPL1, TACC3, JADE1, TTK, DDX3X, CDK5RAP2, DLGAP5, BLM, CDCA5, CDT1, EP300, PSME4, CUL4B, RPA2, NBN, RFWD3, HUS1, PRKDC, DUSP1, MYC, UBE2C, CDC23, BUB1, BUB3, RBL1, SIN3A, PCID2, BRD7, CCNA2, BRCA2, RAD51, TOP2A, NEK7, INCENP, NUMA1, PSRC1, NUSAP1, KNSTRN, SPAG5, KIF23, RACGAP1, PRC1, MCPH1, PPP1R10, DNA2, FANCD2, IL1B, NAA10, MKI67, USP16, ZWINT, MCM3, MCM2, POLA1, POLE, POLE4, POLA2, MCM6, MCM7, MCM5, MCM4, MCM8, PPM1D, NASP, PHF8, SMC1A, KIF22, KIF2C, SGO1, STAG2, STAG1, DSN1, CDCA8, KIFC1, KIF18B, KIF4A, KATNB1, KIF18A, HASPIN, RTEL1, DSCC1, NUF2, CENPW, CENPA, CENPC, NSL1, NCAPD3, NCAPD2, NCAPG, SMC4, SMC2, NCAPG2, NCAPH, CENPH, CENPK, CDC7, STOX1, DTL, TOPBP1, CCNF, PLK2, KNTC1, PLK4, CENPJ, HAUS4, CDC25B, TUBB, CDK2AP2, TPX2, DYNLL1, BRSK1, E2F1, GTSE1, CEP57, CEP70, CEP43, OFD1, CEP192, CEP135, CEP76, PCNT, CEP152, HMMR, CEP78, TUBB4B, APP, CCNB2, MASTL, TIPIN, RBBP8, ATAD5, PSMD14, CDKN1A, SLFN11, CLSPN, TAOK3, FOXM1, KLF4, E2F7, E2F8, ANLN, CDC25A, RRM2, PSMC3, PSME2, PSMF1, PSMA7, PSMD11, CUL1, FBXO7, PSME1, PSMA4,

PSMD6, PSMB8, PSMB10, PSMD8, E2F6, MDM2, TRIM39, RPS27L, PIDD1, RNASEH2B, CNOT8, DBF4, PKMYT1, PLCB1, APPL1, DACT1, AVEN, CDKN2C, ECD, CKS2, CKS1B, CDK14, CCNG1, CCNG2, FBXL3, FBXL12, CCNI, GMNN, PIM2, EIF4EBP1, CUL7, KIF20B, KIF11, CCSAP, KIF20A, ECT2, CEP85, STIL, POC1A, DLG1, EME2, MDC1, INTS3, FBXW5, EGF, DRG1, CAV2, ZWILCH, MAP3K20, DDIAS, HRAS, ORC2, ORC6, MCM10, ORC5, TYMS, UBE2S, FBXL15, TAF2, MELK, GSPT1, PPAT, BCAT1, IQGAP3, DHFRP1, ID4, ZNF324, XRCC2, BIRC5, CKAP2, CEP55, JTB, STMN1, MAP4, XIAP, AAAS, TUBGCP3, TUBGCP2, TUBGCP5, WDR62, TBCE, EML1, LIG1, VCP, SPC25, SAPCD2, SNX9, RASA1, STAMBP, ANKLE2, PTPA, MYBL1, BRCC3, NPM1, PAXIP1, TAF1, DDX11, KAT2B, USP51, BRIP1, TRIM37, EPC1, CENPX, SLX4, POLD1, ESCO2, FANCM, MSH3, RFC2, RFC3, RAD54B, SMARCAD1, KNL1, CENPQ, TIMELESS, NEK10, DDRGK1, PCLAF, CALM2, KLHL21, NUBP1, CEP295, CEP120, ASPM, NSUN2, MAP2K1, GAS2, NUDT16, DACH1, BARD1, FIGNL1, SSTR5, CBX5, PPP2R5B, XPO1, GATA6, MLXIPL, NOTCH1, CDC123, PPP2R3B, TBRG1, RRAGB, IRF1, TP53INP1, CAB39L, INHA, WDR6, PRR11, HEPACAM2, NIN, C2CD3, SSX2IP, CNTLN, SASS6, FANCA, PSMC3IP, PHGDH
**Mitotic cell cycle**
CDK1, CDC6, AURKB, NEK2, CENPF, PCNA, TP53, BRCA1, CHEK1, ATM, GEN1, CDC45, CCNE1, CCNE2, CCNB1, BUB1B, CDC20, RB1, FBXO5, PLK1, AURKA, CENPE, NDC80, SPDL1, MAD1L1, MAD2L1, KIF14, ESPL1, TACC3, JADE1, TTK, DDX3X, CDK5RAP2, DLGAP5, BLM, CDCA5, CDT1, EP300, PSME4, CUL4B, RPA2, NBN, RFWD3, HUS1, PRKDC, DUSP1, MYC, UBE2C, CDC23, BUB1, BUB3, RBL1, SIN3A, PCID2, BRD7, CCNA2, BRCA2, RAD51, TOP2A, NEK7, INCENP, NUMA1, PSRC1, NUSAP1, KNSTRN, SPAG5, KIF23, RACGAP1, PRC1, MCPH1, PPP1R10, DNA2, FANCD2, IL1B, NAA10, MKI67, USP16, ZWINT, MCM3, MCM2, POLA1, POLE, POLE4, POLA2, MCM6, MCM7, MCM5, MCM4, MCM8, PPM1D, NASP, PHF8, SMC1A, KIF22, KIF2C, SGO1, STAG2, STAG1, DSN1, CDCA8, KIFC1, KIF18B, KIF4A, KATNB1, KIF18A, HASPIN, RTEL1, DSCC1, NUF2, CENPW, CENPA, CENPC, NSL1, NCAPD3, NCAPD2, NCAPG, SMC4, SMC2, NCAPG2, NCAPH, CENPH, CENPK, CDC7, STOX1, DTL, TOPBP1, CCNF, PLK2, KNTC1, PLK4, CENPJ, HAUS4, CDC25B, TUBB, CDK2AP2, TPX2, DYNLL1, BRSK1, E2F1, GTSE1, CEP57, CEP70, CEP43, OFD1, CEP192, CEP135, CEP76, PCNT, CEP152, HMMR, CEP78, TUBB4B, APP, CCNB2, MASTL, TIPIN, RBBP8, ATAD5, PSMD14, CDKN1A, SLFN11, CLSPN, TAOK3, FOXM1, KLF4, E2F7, E2F8, ANLN, CDC25A, RRM2, PSMC3, PSME2, PSMF1, PSMA7, PSMD11, CUL1, FBXO7, PSME1, PSMA4, PSMD6, PSMB8, PSMB10, PSMD8, E2F6, MDM2, TRIM39, RPS27L, PIDD1, RNASEH2B, CNOT8, DBF4, PKMYT1, PLCB1, APPL1, DACT1, AVEN, CDKN2C, ECD, CKS2, CKS1B, CDK14, CCNG1, CCNG2, FBXL3, FBXL12, CCNI, GMNN, PIM2, EIF4EBP1, CUL7, KIF20B, KIF11, CCSAP, KIF20A, ECT2, CEP85, STIL, POC1A, DLG1, EME2, MDC1, INTS3, FBXW5, EGF, DRG1, CAV2, ZWILCH, MAP3K20, DDIAS, HRAS, ORC2, ORC6, MCM10, ORC5, TYMS, UBE2S, FBXL15, TAF2, MELK, GSPT1, PPAT, BCAT1, IQGAP3, DHFRP1, ID4, ZNF324, XRCC2, BIRC5, CKAP2, CEP55, JTB, STMN1, MAP4, XIAP, AAAS, TUBGCP3, TUBGCP2, TUBGCP5, WDR62, TBCE, EML1, LIG1, VCP, SPC25, SAPCD2, SNX9, RASA1, STAMBP, ANKLE2, PTPA, MYBL1, SIRT1, YEATS4, DUSP3, NUP214, SKA3, SKA1, TUBA1B, CLIP1, KIF15, TBCD, TUBB6, WDHD1, RRM1, PBK
**Chromosome organization**
CDK1, CDC6, AURKB, NEK2, CENPF, PCNA, TP53, BRCA1, CHEK1, ATM, GEN1, CDC45, CCNE1, CCNE2, CCNB1, BUB1B, CDC20, RB1, FBXO5, PLK1, AURKA, CENPE, NDC80, SPDL1, MAD1L1, MAD2L1, KIF14, ESPL1, TACC3, JADE1, TTK, DDX3X, CDK5RAP2, DLGAP5, BLM, CDCA5, CDT1, EP300, PSME4, CUL4B, RPA2, NBN, RFWD3, HUS1, PRKDC, DUSP1, MYC, UBE2C, CDC23, BUB1, BUB3, RBL1, SIN3A, PCID2, BRD7, CCNA2, BRCA2, RAD51, TOP2A, NEK7, INCENP, NUMA1, PSRC1, NUSAP1, KNSTRN, SPAG5, KIF23, RACGAP1, PRC1, MCPH1, PPP1R10, DNA2, FANCD2, IL1B, NAA10, MKI67, USP16, ZWINT, MCM3, MCM2, POLA1, POLE, POLE4, POLA2, MCM6, MCM7, MCM5, MCM4, MCM8, PPM1D, NASP, PHF8, SMC1A, KIF22, KIF2C, SGO1, STAG2, STAG1, DSN1, CDCA8, KIFC1, KIF18B, KIF4A, KATNB1, KIF18A, HASPIN, RTEL1, DSCC1, NUF2, CENPW, CENPA, CENPC, NSL1, NCAPD3, NCAPD2, NCAPG, SMC4, SMC2, NCAPG2, NCAPH, CENPH, CENPK, BRCC3, NPM1, PAXIP1, TAF1, DDX11, KAT2B, USP51, BRIP1, TRIM37, EPC1, CENPX, SLX4, POLD1, ESCO2, FANCM, MSH3, RFC2, RFC3, RAD54B, SMARCAD1, KNL1, CENPQ, SIRT1, YEATS4, H2AX, FBXO4, ATR, COPS5, ACTB, HCFC1, HELLS, MIS18A, SMARCB1, CHAF1A, CHAF1B, EXO1, UHRF1, CHTF18, ERCC6L, OIP5, MIS18BP1, SUV39H1, HJURP, SUV39H2, EID1, ZMYND11, RAD18, TCP1, CCT4, CCT5, CCT3, MAP1S, PHF1, LEO1, HDAC6, ATXN7, KAT5, SMC6, RNF168, ACTR5, MCRS1, ACTL6A, INO80C, EPC2, NHP2, SMARCA1, N6AMT1, DKC1, EXOSC10, MBD3, NOP10, IGHMBP2, POLQ, MCM9, GINS4, GINS2, SETX, XRCC6, XRCC1, KMT5B, EMSY, HMGB1, HMGB2, PARP10, DTX3L, HMGN1, EYA1, DEK, KDM2A, DNMT1, TINF2, H1-10, APOBEC1, PARN, XRN1, KDM1B, TOX, NAP1L2, ASH2L, ING5, BRPF3, DPY30, MEAF6, ZNHIT1, SMARCC1, CREBBP, GTF2B, KAT6B, SMARCC2, OGT, KDM3A, JADE2, BRD8, CXXC1, BRMS1, ING3, KDM5D, KDM4A, MSL3, SIN3B, BRD9, SMYD2, CDAN1, CENPN, CENPM, CENPI, CENPP, MACROH2A2, HAT1, JAK2, CAMK2D, PINK1, ATAD2, PKN1, GPX4, THAP7, ISL1, MCM3AP, H2BC5, NAP1L3, ASF1B, UBN1, TCF7L1, PPM1F, NELFE, GLYR1, NUP155, FAM172A, NR1H4, H2AZ1, FOXA3, CDCA4, ELOF1, HIRIP3
**Regulation of cell cycle**
CDK1, CDC6, AURKB, NEK2, CENPF, PCNA, TP53, BRCA1, CHEK1, ATM, GEN1, CDC45, CCNE1, CCNE2, CCNB1, BUB1B, CDC20, RB1, FBXO5, PLK1, AURKA, CENPE, NDC80, SPDL1, MAD1L1, MAD2L1, KIF14, ESPL1, TACC3, JADE1, TTK, DDX3X, CDK5RAP2, DLGAP5, BLM, CDCA5, CDT1, EP300, PSME4, CUL4B, RPA2, NBN, RFWD3, HUS1, PRKDC, DUSP1, MYC, UBE2C, CDC23, BUB1, BUB3, RBL1, SIN3A, PCID2, BRD7, CCNA2, BRCA2, RAD51, TOP2A, NEK7, INCENP, NUMA1, PSRC1, NUSAP1, KNSTRN, SPAG5, KIF23, RACGAP1, PRC1, MCPH1, PPP1R10, DNA2, FANCD2, IL1B, NAA10, MKI67, USP16, ZWINT, CDC7, STOX1, DTL, TOPBP1, CCNF, PLK2, KNTC1, PLK4, CENPJ, HAUS4, CDC25B, TUBB, CDK2AP2, TPX2, DYNLL1, BRSK1, E2F1, GTSE1, CEP57, CEP70, CEP43, OFD1, CEP192, CEP135, CEP76, PCNT, CEP152, HMMR, CEP78, TUBB4B, APP, CCNB2, MASTL, TIPIN, RBBP8, ATAD5, PSMD14, CDKN1A, SLFN11, CLSPN, TAOK3, FOXM1, KLF4, E2F7, E2F8, ANLN, CDC25A, RRM2, PSMC3, PSME2, PSMF1, PSMA7, PSMD11, CUL1, FBXO7, PSME1, PSMA4, PSMD6, PSMB8, PSMB10, PSMD8, E2F6, MDM2, TRIM39, RPS27L, PIDD1, RNASEH2B, CNOT8, DBF4, PKMYT1, PLCB1, APPL1, DACT1, AVEN, CDKN2C, ECD, CKS2, CKS1B, CDK14, CCNG1, CCNG2, FBXL3, FBXL12, CCNI, GMNN, PIM2, EIF4EBP1, CUL7, KIF20B, KIF11, CCSAP, KIF20A, ECT2, CEP85, STIL, POC1A, DLG1, EME2, MDC1, INTS3, FBXW5, EGF, DRG1, CAV2, ZWILCH, MAP3K20, DDIAS, HRAS, BRCC3, NPM1, PAXIP1, TAF1, DDX11, KAT2B, USP51, BRIP1, TRIM37, EPC1, TIMELESS, NEK10, DDRGK1, PCLAF, CALM2, KLHL21, NUBP1, CEP295, CEP120, ASPM, NSUN2, MAP2K1, GAS2, NUDT16, DACH1, BARD1, FIGNL1, SSTR5, CBX5, PPP2R5B, XPO1, GATA6, MLXIPL, NOTCH1, CDC123, PPP2R3B, TBRG1, RRAGB, IRF1, TP53INP1,

CAB39L, INHA, WDR6, PRR11, SIRT1, DUSP3, NUP214, H2AX, FBXO4, ATR, COPS5, ACTB, HCFC1, PRKACB, THOC1, LIN9, TRIM21, CRY1, JUN, BIRC2, NR4A1, E2F2, CCNDBP1, LIN54

Upregulated genes:

**Cell projection organization**
LRP1, SRC, FLOT1, CNR1, DNM2, RAB11A, SNAPIN, SYT3, CDH1, RAB13, RAB10, CNTN1, PICALM, FZD4, MT3, TMEM108, MAPT, PTPRS, KIF1A, PLD1, CORO1C, SMO, EZR, SDC4, RAB17, NTRK1, ROR2, AREG, EPHA8, UNC5B, FOLR1, NFASC, RND2, CLMN, RHOQ, TCIRG1, EMP2, RAB34, RHOV, RCC2, ABCD2, CDC42, AKT1, MAPK8IP2, STX1B, NRXN1, CACNG7, SHANK1, PRKCI, HAP1, PPP3CA, SRCIN1, PACSIN1, ATP1B2, ROR1, TNIK, RTN4, CYFIP2, SYNGAP1, IQSEC1, DBN1, ZMYND8, MAP1A, KIF5A, SLC12A5, AGRN, CTNNA2, SPOCK1, CNTNAP1, WHRN, IGSF9, PALLD, SAMD14, SEMA3F, SEMA4B, PHACTR1, BCL11A, LAMA5, WASF3, NDE1, DMTN, PALM, ATP1A3, DYNLL2, PTPN11, AGT, RASGRF1, PAK1, VEGFA, PIK3R1, NDRG4, SPP1, RAB29, SCN1B, RUFY3, TRIM46, AMIGO1, ATP8A2, MICALL2, EFNA1, VANGL2, ROBO1, KIDINS220, HDAC2, GAB1, PIK3CA, SHC1, MACF1, CUX2, YTHDF1, PTK7, TNFRSF12A, NGEF, STMN3, NIBAN2, KREMEN1, CTSZ, TRAK1, L1CAM, CRMP1, KNDC1, RNF6, ATAT1, PTPRF, KIF5C, BBS4, ARL3, EPHA1, LHFPL5, FLRT1, DPYSL5, PDZD7, ATP8B1, USH1G, SEMA6C, SEMA6B, SEMA4G, LINGO1, ITGA6, PLK5, LRTM2, VASP, B4GALT6, LYPLA2, S100A6, PTPRM, ARTN, RPS6KA5, NYAP1, TBC1D23, VASH2, ALKBH1, F2RL1, RAB25, ARAP1, FAM110C, LGMN, NECTIN2, WTIP, PKHD1, VAV2, NME5, JHY, TUBB4A, ESPN, RREB1, AKIRIN1, RILPL1, RILPL2, CCDC39, ARFIP2, CROCC, SEPTIN9, DZIP1, TNPO1, WDR60, CAPZB, CDC42EP4, CDC42EP5, PARVB, PLEK2, INPPL1, CARMIL2, CCDC65, DNAI1, FHDC1, KCTD17, CNTROB, MTSS1, AK7, CDC14B, ABLIM1, CEP126, DNAI2, CCDC114, CEP41, CFAP70, BBOF1, UNC119B
**Whole membrane**
LRP1, SRC, FLOT1, CNR1, DNM2, RAB11A, SNAPIN, SYT3, CDH1, RAB13, RAB10, CNTN1, PICALM, FZD4, MT3, TMEM108, MAPT, PTPRS, KIF1A, PLD1, CORO1C, SMO, EZR, SDC4, RAB17, NTRK1, ROR2, AREG, EPHA8, UNC5B, FOLR1, NFASC, RND2, CLMN, RHOQ, TCIRG1, EMP2, RAB34, RHOV, RCC2, ABCD2, TH, GNAI2, SEPTIN4, HIP1, STX1A, SYT7, RAB3B, SYT12, ZDHHC2, ITPR1, WFS1, SYP, SV2A, RAB26, CLN3, CNIH2, ATAD1, LAMP1, CBARP, RAB12, SYNGR3, SEPTIN8, ATP2B1, KCNH1, CLSTN1, PRKAR1A, SNAP23, GAD1, SYNGR1, OTOF, GNB1, SLC12A4, VDAC1, SYPL1, STX7, ZNRF2, INPP4A, APPL2, MYO6, ACSL3, FZD7, PSAP, ADGRG1, FOXO3, SUN2, SPHK2, RAP1B, TNFRSF1A, PTPRJ, TRAF2, HYAL2, BID, SNX4, CEACAM1, ITGAV, KCNQ1, DMPK, RAB7A, STOM, RAB31, HYAL3, LDLRAP1, REEP6, SREBF2, SAR1B, C11orf65, SCARB1, STIM1, CD24, HPSE, SLC44A2, TFRC, RRAGD, BMF, HBEGF, TICAM1, LCK, LITAF, TSPAN14, RHOF, ADGRG3, NPRL2, DEPDC5, CBLC, ADGRE5, ENPEP, ECE2, ITGA2B, CAVIN1, CYB5R1, ORMDL3, ALDH3B1, MAGT1, STEAP3, MOSPD2, PRCP, STK10, RABEPK, PHACTR2, APLP2, MLEC, MANBA, CYSTM1, MYO1D, HSPA9, FAM210B, PLEKHB2, FNDC5, AP1S3, SKAP1, VPS13A, WIPI1, AP1G2, GGA2, KDELR3, BICD2, TSPAN15, RAB22A, RABEP1, RASSF9, VPS37B, VPS37D, RABGEF1, MARCHF5, VPS13C, TOMM20, ATG9B, SLC7A5, ACACB, STAT6, PLPP2, SLC3A2, AQP4, MREG, NECAP2, ABCD4, ACSL6, PEX11A, SLC26A11, SLC9A7, ATP6V0B, PRAF2, PARM1, MR1, PMEPA1, PLIN3, MARCHF9, CD164, APH1B, MFSD12, IZUMO1, TMEM184A, MARCHF2, AIFM2, LMBRD1, CCDC136, CYB561, MAP1LC3B, EXD2, SNN, BRI3BP, ARMCX6, ARMCX2, TMEM35A
**Synapse**
LRP1, SRC, FLOT1, CNR1, DNM2, RAB11A, SNAPIN, SYT3, CDH1, RAB13, RAB10, CNTN1, PICALM, FZD4, MT3, TMEM108, MAPT, PTPRS, KIF1A, PLD1, CORO1C, CDC42, AKT1, MAPK8IP2, STX1B, NRXN1, CACNG7, SHANK1, PRKCI, HAP1, PPP3CA, SRCIN1, PACSIN1, ATP1B2, ROR1, TNIK, RTN4, CYFIP2, SYNGAP1, IQSEC1, DBN1, ZMYND8, MAP1A, KIF5A, SLC12A5, AGRN, CTNNA2, SPOCK1, CNTNAP1, WHRN, IGSF9, PALLD, SAMD14, SEMA3F, SEMA4B, PHACTR1, BCL11A, LAMA5, WASF3, NDE1, DMTN, PALM, ATP1A3, DYNLL2, TH, GNAI2, SEPTIN4, HIP1, STX1A, SYT7, RAB3B, SYT12, ZDHHC2, ITPR1, WFS1, SYP, SV2A, RAB26, CLN3, CNIH2, ATAD1, LAMP1, CBARP, RAB12, SYNGR3, SEPTIN8, ATP2B1, KCNH1, CLSTN1, PRKAR1A, SNAP23, GAD1, SYNGR1, OTOF, GNB1, SLC12A4, VDAC1, SYPL1, STX7, ZNRF2, INPP4A, STAT3, NR1D1, CLU, ARHGEF2, ETV5, MDGA1, SDK2, ADORA1, MAPK14, FGA, ARRB2, SLC4A8, NLGN2, RIMS3, SHISA7, HOMER3, KCNC2, HRH3, CACNA1G, ACHE, ASIC1, DNM1, UNC119, MICAL1, LGI3, HPCA, KCNC1, KCND1, HCN3, CABP1, MKLN1, KCNJ4, GNB5, SLC8A2, PLEKHG5, RASD2, MAPK8IP1, PJA2, MADD, CTBP2, IGSF9B, MPP2, CRHR2, PSD2, SLC29A1, NAAA, PPFIA3, PDYN, APC2, IL1RAP, GRID2IP, PLCB3, KCNK3, ANXA9, SST, PLAT, LY6H, GJD2, MYCBPAP, CANX, OLFM2, SEPTIN3, GABBR1, CPEB1, FBXO2, RGS17, RPL38, RPL3, SLC29A4, LRFN1, SLC29A2, BCAN, PPFIBP1, TUFM, COL4A5, PLEKHA5, SYNC, FRRS1L
**Neurogenesis**
LRP1, SRC, FLOT1, CNR1, DNM2, RAB11A, SNAPIN, SYT3, CDH1, RAB13, RAB10, CNTN1, PICALM, FZD4, MT3, TMEM108, MAPT, PTPRS, KIF1A, SMO, EZR, SDC4, RAB17, NTRK1, ROR2, AREG, EPHA8, UNC5B, FOLR1, NFASC, RND2, CLMN, CDC42, AKT1, MAPK8IP2, STX1B, NRXN1, CACNG7, SHANK1, PRKCI, HAP1, PPP3CA, SRCIN1, PACSIN1, ATP1B2, ROR1, TNIK, RTN4, CYFIP2, SYNGAP1, IQSEC1, DBN1, ZMYND8, MAP1A, KIF5A, SLC12A5, AGRN, CTNNA2, SPOCK1, CNTNAP1, WHRN, IGSF9, PALLD, SAMD14, SEMA3F, SEMA4B, PHACTR1, BCL11A, LAMA5, WASF3, NDE1, PTPN11, AGT, RASGRF1, PAK1, VEGFA, PIK3R1, NDRG4, SPP1, RAB29, SCN1B, RUFY3, TRIM46, AMIGO1, ATP8A2, MICALL2, EFNA1, VANGL2, ROBO1, KIDINS220, HDAC2, GAB1, PIK3CA, SHC1, MACF1, CUX2, YTHDF1, PTK7, TNFRSF12A, NGEF, STMN3, NIBAN2, KREMEN1, CTSZ, TRAK1, L1CAM, CRMP1, KNDC1, RNF6, ATAT1, PTPRF, KIF5C, BBS4, ARL3, EPHA1, LHFPL5, FLRT1, DPYSL5, PDZD7, ATP8B1, USH1G, SEMA6C, SEMA6B, SEMA4G, LINGO1, ITGA6, PLK5, LRTM2, VASP, B4GALT6, LYPLA2, S100A6, PTPRM, ARTN, RPS6KA5, NYAP1, TBC1D23, VASH2, ALKBH1, TH, APPL2, MYO6, ACSL3, FZD7, PSAP, ADGRG1, FOXO3, SUN2, STAT3, NR1D1, CLU, ARHGEF2, ETV5, MDGA1, SDK2, TGFB1, GAS6, ERBB3, CHD7, CDON, CHD5, ZMIZ1, GDF11, YAP1, RORA, SLC44A4, RARA, SOCS2, GNAT1, AGBL4, PRDM1, KCTD11, DNMT3B, HMG20B, OTP, DTX1, ZFHX3, HMG20A, ZFHX2, CASZ1, PDE6C, NFE2L1, NRBP2, NGRN, LEMD2, SIRT2, DNER, TMEM98, SOX6, ZNF609, PLAG1, SCRT1, IGSF10, GPR157, ASTN1, CHAC1, TEAD3

Regulation of transport
LRP1, SRC, FLOT1, CNR1, DNM2, RAB11A, SNAPIN, SYT3, CDH1, RAB13, RAB10, CNTN1, PICALM, PLD1, SMO, EZR, SDC4, RAB17, RHOQ, TCIRG1, CDC42, AKT1, MAPK8IP2, STX1B, NRXN1, CACNG7, SHANK1, PRKCI, HAP1, PPP3CA, SRCIN1, PACSIN1, ATP1B2, DMTN, PTPN11, AGT, RASGRF1, PAK1, VEGFA, PIK3R1, NDRG4, SPP1, RAB29, SCN1B, RUFY3, TRIM46, AMIGO1, ATP8A2, MICALL2, F2RL1, RAB25, GNAI2, SEPTIN4, HIP1, STX1A, SYT7, RAB3B, SYT12, ZDHHC2, ITPR1, WFS1, SYP, SV2A, RAB26, CLN3, CNIH2, ATAD1, LAMP1, CBARP, RAB12, SYNGR3, SEPTIN8, ATP2B1, KCNH1, APPL2, MYO6, ACSL3, SPHK2, RAP1B, TNFRSF1A, PTPRJ, TRAF2, HYAL2, BID, SNX4, CEACAM1, ITGAV, KCNQ1, DMPK, RAB7A, STOM, RAB31, HYAL3, LDLRAP1, REEP6, SREBF2, SAR1B, C11orf65, SCARB1, STIM1, STAT3, NR1D1, CLU, ADORA1, MAPK14, FGA, ARRB2, SLC4A8, NLGN2, RIMS3, SHISA7, HOMER3, KCNC2, HRH3, CACNA1G, ACHE, ASIC1, DNM1, UNC119, MICAL1, LGI3, HPCA, KCNC1, KCND1, HCN3, CABP1, MKLN1, KCNJ4, GNB5, TGFB1, GAS6, ERBB3, CHD7, TCF7L2, TNFRSF11A, PYCARD, SIRT3, PDX1, CD40, CD81, FKBP1A, ARHGEF5, PEA15, TRPM4, CHGA, HFE, GPR27, PFKFB2, TM7SF3, RNF207, KCNN4, CHCHD10, JPH4, JPH3, OPRL1, TMBIM6, CD22, JSRP1, LRP5, SIRT4, HADH, CACNA2D2, KDM5B, SLC8B1, ABCC8, ACVR1C, HMGN3, G6PC2, FAM3D, PNKD, HCN2, KCNJ5, FGF11, LLGL2, RHBDF1, CACNA1F, AZIN2, TESC, PKDCC, KMT2A, TMEM30B, CRACR2B, RIPOR1, OAZ2, NPEPPS, SIAH3, SLC38A3, KCNQ4, KCNH2, KCNA7, KCNF1, KCNH4, KCNAB3, KCNH3, HSPA2, PACSIN3, CACNA2D4, ANO9, CD151, ANKRD13B, ARPP19, RBM27
